# Algorithms for Multipolar Interval-Valued Neutrosophic Soft Set with Information Measures to Solve Multicriteria Decision-Making Problem

**DOI:** 10.1155/2021/7211399

**Published:** 2021-11-10

**Authors:** Rana Muhammad Zulqarnain, Imran Siddique, Aiyared Iampan, Ebenezer Bonyah

**Affiliations:** ^1^Department of Mathematics, School of Science, University of Management and Technology, Sialkot Campus, Lahore, Pakistan; ^2^Department of Mathematics, School of Science, University of Management and Technology, Lahore 54770, Pakistan; ^3^Department of Mathematics, School of Science, University of Phayao, Mueang, Mae Ka 56000, Phayao, Thailand; ^4^Department of Mathematics Education, Akenten Appiah-Menka University of Skills Training and Entrepreneurial Development, Kumasi 00233, Ghana

## Abstract

Similarity measures (SM) and correlation coefficients (CC) are used to solve many problems. These problems include vague and imprecise information, excluding the inability to deal with general vagueness and numerous information problems. The main purpose of this research is to propose an m-polar interval-valued neutrosophic soft set (mPIVNSS) by merging the m-polar fuzzy set and interval-valued neutrosophic soft set and then study various operations based on the proposed notion, such as AND operator, OR operator, truth-favorite, and false-favorite operators with their properties. This research also puts forward the concept of the necessity and possibility operations of mPIVNSS and also the m-polar interval-valued neutrosophic soft weighted average operator (mPIVNSWA) with its desirable properties. Cosine and set-theoretic similarity measures have been proposed for mPIVNSS using Bhattacharya distance and discussed their fundamental properties. Furthermore, we extend the concept of CC and weighted correlation coefficient (WCC) for mPIVNSS and presented their necessary characteristics. Moreover, utilizing the mPIVNSWA operator, CC, and SM developed three novel algorithms for mPIVNSS to solve the multicriteria decision-making problem. Finally, the advantages, effectiveness, flexibility, and comparative analysis of the developed algorithms are given with the prevailing techniques.

## 1. Introduction

Multicriteria decision-making (MCDM) is an essential condition for decision scientific discipline. The decision-maker should judge the choices stated by the diverse forms of distinguishing perspectives. Though, in quite a lot of situations, it is tough for someone to undertake it because of numerous uncertainties in the data. One is due to lack of expertise or ravishment of policies. Thus, to measure the given disadvantages and thinking tools, a succession of philosophies had been projected. Zadeh introduced the notion of the fuzzy set (FS) [1] to resolve complex problems that contain vagueness and uncertainty. But FS is unable to handle the environment when any expert considers the membership (Mem) grade of any object in the intervals form. To overawed such states, Turksen [2] proposed the idea of interval-valued fuzzy sets (IVFS). Sometimes, decision-makers consider the nonmembership (NMem) value of the object which cannot be processed by FS nor by IVFS. Atanassov [3] settled the concept of intuitionistic fuzzy sets (IFS) to contract above declared complications. The idea proposed by Atanassov involves only underconsidered data as well as Mem and Nmem values. However, the IFS theory is unable to cope with overall incompatibility and inaccurate data. To resolve the challenge of incompatibility and incorrect data, Smarandache [4] planned the theory of NS. Molodtsov [5] presented a universal accurate tool for addressing uncertain environments renowned as soft set (SS).

Maji et al. [6] expanded the concept of SS and proposed fundamental operations with their desired properties. Maji et al. [7] established a decision-making (DM) technique utilizing their developed operations and used it for DM. Ali et al. [8] extended the notion of SS and established some novel operations with their fundamental properties. The authors [9] extended the notion of SS and proved De Morgan's law. The concept of soft matrices has been developed by Cagman and Enginoglu [10]; they also familiarized some basic operations for soft matrices and studied their required possessions. Cagman and Enginoglu [11] protracted the concept of SS with some fundamental operations and discussed their characteristics. Furthermore, a DM approach has been established to solve DM difficulties employing their settled operations. In [12], the author presented some novel operations with their properties. Maji [13] presented the impression of a neutrosophic soft set (NSS) along with necessary operations and possessions. Liu et al. [14] discussed the class of uncertain fractional-order neural networks with external disturbances using adaptive fuzzy control. Broumi [15] extended the notion of NSS and proposed the generalized form of NSS and discussed some fundamental operations. Zulqarnain et al. [16] presented the idea of multipolar neutrosophic soft sets and discussed their desirable properties.

Correlation plays a significant part in statistics as well as engineering science. The joint association of two variable quantities can be utilized to appraise the interdependency of the correlation qualitative analysis. In addition to using probabilistic strategies for noticeably pragmatic engineering science complications, you also can locate various boundaries to probabilistic methods. However, the bodily structure has numerous exceptions, the improvement is challenging, and it is difficult to obtain exact consequences. Thus, due to the wide variety of incomprehensible info, the consequences of probability theory are not able to provide professionals with suitable information. In addition, in natural world concerns, there is not any priggish reason out to deal along with distinguished statistical information. Due to the preliminary limitations, the outcomes of probability theory are not conducive to specialists. So, probability theory is not very adequate to resolve the insecurity explicit in the data. Several assessors around the world have prearranged and suggested different strategies to solve anxiety-related complications. Wang et al. [17] developed a decision-making (DM) technique utilizing CC for single valued neutrosophic soft sets (SVNSs). Zulqarnain et al. [18] established the generalized neutrosophic TOPSIS to solve MCDM issues. Hashmi et al. [19] merged two existing theories such as multipolar FS and neutrosophic set and proposed a multipolar neutrosophic set. Zulqarnain et al. [20] introduced the CC for Pythagorean fuzzy soft set and developed the TOPSIS method based on CC for supplier selection in green supply chain management.

Gerstenkorn and Mańko [21] proposed a method for IFS correlation, and they also presented the characteristic coefficients. Yu [22] proposed the CC for fuzzy numbers to measure the relation among fuzzy numbers. Chiang and Lin [23] have developed a way to test the CC of fuzzy data. Zulqarnain et al. [24] proposed the TOPSIS technique to resolve multiattribute decision-making complications constructed on CC for the interval-valued intuitionistic fuzzy soft set (IVIFSS). Hung and Wu [25] intended a novel technique to measure the center of gravity for IFS and gave the presented method to interval-valued IFS. Bustince and Burillo [26] presented the relationship between IVIFS and CC, proving the decomposition theorem for IVIFS. Hong [27] and Mitchell [28] developed the CC for IFS and IVIFS. Zulqarnain et al. [29] protracted the AOs and CC foe intuitionistic fuzzy hypersoft set, and they also constructed a TOPSIS method utilizing their proposed CC. Zulqarnain et al. [30] presented a DM technique based on CC for the interval-valued neutrosophic hypersoft set. Samad et al. [31] utilized the TOPSIS approach for the selection of the most suitable hand sanitizer in this pandemic under the neutrosophic hypersoft scenario.

In the past few years, quite a lot of mathematicians have progressed numerous methodologies such as similarity measures, CC, and aggregation operators (AOs) along with their applications in DM. Harish [32] offered the weighted cosine SM of IFS. Additionally, they built the MCDM approach under his planned methodology and used the progressed strategies for information processing and diagnosis. Garg and Kumar [33] recommended some novel SM to evaluate the relative strength of IFS. Liu et al. [34] studied the synchronization for a class of indeterminate fractional-order neural networks focusing on peripheral instabilities and distressed scheme parameters. Peng and Garg [35] proposed a variety of Pythagorean fuzzy set (PFS) similarity measures with multiple parameters. The notion of the m-polar neutrosophic soft set (mPNSS) was developed by Saeed et al. [36]. Liu et al. [37] studied synchronization problematic of fractional-order chaotic schemes through input capacity and indefinite exterior trouble using adaptive fuzzy control. Zulqarnain et al. [38] offered a DM technique based on a score matrix to solve multiattribute decision-making (MADM) problems for neutrosophic hypersoft matrices. Riaz et al. [39] developed an MCDM method for soft multiset, and they also presented the soft multiset topology AOs. Zulqarnain et al. [40] proposed the integrated neutrosophic TOPSIS to resolve MCDM concerns.

In this epoch, specialists believe that real life is touching the track of multipolarization. Therefore, there is no distrust that the multipolarization of information has played a significant part in the prosperity of many arenas of science and technology. In neurobiology, multipolar neurons in the brain accumulate a lot of info from other neurons. In the whole manuscript, the motivation for expanding and mixing this research work is gradually given. We prove that under any appropriate circumstances, different hybrid structures comprehending FS will be transformed into distinct privileges of mPIVNSS. This study will be the utmost versatile form that can be used to merge data in daily life complications. The organization of the current research is such as follows: some basic concepts are presented in Section 2, which helps us to construct the structure of the following study. In Section 3, we propose a novel idea of mPIVNSS by combining the m-polar fuzzy set (mPFS) and interval-valued neutrosophic soft set, its properties, and operations. In Section 4, the concepts and properties of CC and WCC are presented, and the decision-making method is established based on CC. In Section 5, the multipolar interval-valued neutrosophic soft weighted aggregation (mPIVNSWA) operator and two different types of similarity measures with their decision-making methods are developed. In Section 6, we use the developed techniques for DM and present a numerical example. In Section 7, a brief comparison between our developed approach and existing techniques is provided. Also, superiority, practicality, and flexibility have been introduced in the same section.

## 2. Preliminaries

In the following section, we recalled some fundamental concepts which help us to construct the structure of the following study.

**Definition 1 .**

(See [4]).

Let *𝒰* be a universe, and *𝒜* be an NS on *𝒰* defined as *𝒜*={<*u*, *u*_*𝒜*_(*u*), *v*_*𝒜*_(*u*), *w*_*𝒜*_(*u*) > :*u* ∈ *𝒰*}, where *u*, *v*, *w*: *𝒰*⟶]0^−^, 1^+^[ and 0^−^ ≤ *u*_*𝒜*_(*u*)+*v*_*𝒜*_(*u*)+*w*_*𝒜*_(*u*) ≤ 3^+^.

**Definition 2 .**

(See [19]).

Let *𝒰* be the universal set and *℘*_*ℜ*_ is said to be the multipolar neutrosophic set if ${℘}_{ℜ}=\left\{\begin{array}{c} \left(u,u_{\alpha }\left(u\right)\;,v_{\alpha }\left(u\right)\;,w_{\alpha }\left(u\right)\right):u\in U,\alpha =\;1,\;2,\;3,\ldots ,\;m \end{array}\;\right\}$, where *u*_*α*_(*u*), *v*_*α*_(*u*), and *w*_*α*_(*u*) represent the truthiness, indeterminacy, and falsity, respectively, *u*_*α*_(*u*), *v*_*α*_(*u*), *w*_*α*_(*u*)⊆[0,1] and 0 ≤ *u*_*α*_(*u*)+*v*_*α*_(*u*)+*w*_*α*_(*u*) ≤ 3, for all *α*=1,2,…, *m*; and *u* ∈ *𝒰*.

**Definition 3 .**

(See [5]).

Let *𝒰* be the universal set and *ℰ* be the set of attributes concerning *𝒰*. Let *𝒫*(*𝒰*) be the power set of *𝒰* and *𝒜*⊆*ℰ*. A pair (*ℱ*, *𝒜*) is called an SS over *𝒰* and its mapping is given as

(1) $$\begin{array}{c} F:A⟶P\left(U\right). \end{array}$$

It is also defined as

(2) $$\begin{array}{c} \left(F,A\right)=\left\{F\left(e\right)\in P\left(U\right):e\in E,F\left(e\right)=\;\emptyset \text{ if }e\neq A\right\}. \end{array}$$

**Definition 4 .**

(See [13]).

Let *𝒰* be the universal set and *ℰ* be the set of attributes concerning *𝒰*. Let *𝒫*(*𝒰*) be the set of neutrosophic sets over *𝒰* and  *𝒜*⊆*ℰ*. A pair (*ℱ*, *𝒜*) is called an NSS over *𝒰*, and its mapping is given as

(3) $$\begin{array}{c} F:A⟶P\left(U\right). \end{array}$$

**Definition 5 .**

(See [41]).

Let *𝒰* be a universal set; then, the interval-valued neutrosophic set can be expressed by the set *𝒜*={*u*, (*u*_*𝒜*_(*u*), *v*_*𝒜*_(*u*), *w*_*𝒜*_(*u*)) : *u* ∈  *𝒰*}, where *u*_*𝒜*_, *v*_*𝒜*_, and *w*_*𝒜*_ are truth, indeterminacy, and falsity membership functions for *𝒜*, respectively, *u*_*𝒜*_, *v*_*𝒜*_, and *w*_*𝒜*_⊆[0,1] for each *u* ∈ *𝒰*, where

(4) $$\begin{array}{c} u_{A}\left(u\right)=\left[u_{A}^{L}\;\left(u\right),u_{A}^{U}\;\left(u\right)\right],\\ v_{A}\left(u\right)=\left[v_{A}^{L}\;\left(u\right),v_{A}^{U}\left(u\right)\right],\\ w_{A}\left(u\right)=\left[w_{A}^{L}\;\left(u\right),w_{A}^{U}\left(u\right)\right]. \end{array}$$

For each point, *u* ∈ *𝒰*, 0 ≤ *u*_*𝒜*_(*u*)+*v*_*𝒜*_(*u*)+*w*_*𝒜*_(*u*) ≤ 3, and IVN (*𝒰*) represent the family of all interval-valued neutrosophic sets on *𝒰*.

**Definition 6 .**

(See [42]).

Let *𝒰* be a universe of discourse and *ℰ* be a set of attributes, and an m-polar neutrosophic soft set (mPNSS) *℘*_*ℜ*_ over *𝒰* defined as

(5) $$\begin{array}{c} {℘}_{R}=\left\{\begin{array}{c} \left(e,\left\{\left(u,u_{\alpha }\left(u\right),v_{\alpha }\left(u\right),w_{\alpha }\left(u\right)\right):u\in U,\alpha =\;1,\;2,\;3,\ldots ,m\right\}\right):e\in E \end{array}\right\}, \end{array}$$

where *u*_*α*_(*u*), *v*_*α*_(*u*), and *w*_*α*_(*u*) represent the truthiness, indeterminacy, and falsity, respectively, *u*_*α*_(*u*), *v*_*α*_(*u*), *w*_*α*_(*u*)⊆[0,1] and 0 0 ≤ *u*_*α*_(*u*)+*v*_*α*_(*u*)+*w*_*α*_(*u*) ≤ 3, for all *α*=1,2,3,…, *m*; *e* ∈ *ℰ* and *u* ∈ *𝒰*. Simply an m-polar neutrosophic number (mPNSN) can be expressed as *℘*={〈*u*_*α*_, *v*_*α*_,  *w*_*α*_〉}, where 0 ≤ *u*_*α*_+*v*_*α*_+*w*_*α*_ ≤ 3 and *α*=1,2,3,…, *m*.

**Definition 7 .**

(See [43]).

Let *𝒰* be a universe of discourse and *ℰ* be a set of attributes, and IVNSS *℘*_*ℜ*_ over *𝒰* defined as

(6) $$\begin{array}{c} {℘}_{R}=\left\{\begin{array}{c} \left(e,\left\{\left(u,u_{R}\left(u\right),v_{R}\left(u\right),w_{R}\left(u\right)\right):u\in U,\alpha =\;1,\;2,\;3,\ldots ,\;m\right\}\right):e\;\in \;E \end{array}\right\}, \end{array}$$

where *u*_*ℜ*_(*u*)=[*u*_*ℜ*_^*ℓ*^(*u*), *u*_*ℜ*_^*𝔲*^(*u*)], *v*_*ℜ*_(*u*)=[*v*_*ℜ*_^*ℓ*^(*u*), *v*_*ℜ*_^*𝔲*^(*u*)], and *w*_*ℜ*_(*u*)=[*w*_*ℜ*_^*ℓ*^(*u*), *w*_*ℜ*_^*𝔲*^(*u*)] represents the interval truthiness, indeterminacy, and falsity, respectively, *u*_*ℜ*_(*u*), *v*_*ℜ*_(*u*), *w*_*ℜ*_(*u*)⊆[0,  1] and 0 ≤ *u*_*ℜ*_^*𝔲*^(*u*)+*v*_*ℜ*_^*𝔲*^(*u*)+*w*_*ℜ*_^*𝔲*^(*u*) ≤ 3, for each *e* ∈ *ℰ* and *u* ∈ *𝒰*.

## 3. Multipolar Interval-Valued Neutrosophic Soft Set with Aggregate Operators and Properties

The idea of the m-polar fuzzy set (mPFS) was developed by Chen et al. [44] in 2014, capable of addressing data with ambiguity along with vagueness multipolar information. Smarandache [45] presented the tripolar, multipolar neutrosophic sets as well as their graph in 2016. The membership degree range of mPFS is in the interval [0,1]^*m*^, representing *m* criteria of the object, but mPFS cannot handle indeterminacy and falsity objects. NS bargains with truth, falsity, one any choice specifications containing indeterminacy, but are not able to deal with multicriteria, multiple sources, and multiple polarities information fusion of possible choices. To conquer this question, Deli et al. [42] combined the concept of the m-polar neutrosophic set and SS and introduced a new model of mPNSS. The developed mPNSS can deal with *m* criteria for each alternative. mPNSS is an extension of the bipolar NSS which was introduced by Ali et al. [46]. Deli [43] established the IVNSS which was the combination of IVNS [41] and SS [5]. We build some basic concepts of mPNSS and extend the mPNSS to mPIVNSS with various operations and properties.

**Definition 8 .**

Let *𝒰* be a universe of discourse and *ℰ* be a set of attributes, an m-polar interval-valued neutrosophic soft set (mPIVNSS) *℘*_*ℜ*_ over *𝒰* defined as

(7) $$\begin{array}{c} {℘}_{ℜ}=\left\{\left(e,\left\{\left(u,u_{\alpha }\left(u\right),v_{\alpha }\left(u\right),w_{\alpha }\left(u\right)\right):u\in U,\alpha =1,2,3,\ldots ,m\right\}\right):e\in E\right\}, \end{array}$$

where *u*_*α*_(*u*)=[*u*_*α*_^*ℓ*^(*u*), *u*_*α*_^*𝔲*^(*u*)], *v*_*α*_(*u*)=[*v*_*α*_^*ℓ*^(*u*), *v*_*α*_^*𝔲*^(*u*)], and *w*_*α*_(*u*)=[*w*_*α*_^*ℓ*^(*u*), *w*_*α*_^*𝔲*^(*u*)] represent the interval truthiness, indeterminacy, and falsity, respectively; *u*_*α*_(*u*), *v*_*α*_(*u*), *w*_*α*_(*u*)⊆[0,  1] and 0 ≤ *u*_*α*_^*𝔲*^(*u*)+*v*_*α*_^*𝔲*^(*u*)+*w*_*α*_^*𝔲*^(*u*) ≤ 3 for all *α*=1,2,3,…, *m*; *e* ∈ *ℰ* and *u* ∈ *𝒰*. Simply, an m-polar interval-valued neutrosophic soft number (mPIVNSN) can be expressed as $℘=\left\{\begin{array}{c} \left[u_{\alpha }^{\ell }\left(u\right),u_{\alpha }^{𝔲}\left(u\right)\right],\left[v_{\alpha }^{\ell }\left(u\right),v_{\alpha }^{𝔲}\left(u\right)\right],\;\left[w_{\alpha }^{\ell }\left(u\right),w_{\alpha }^{𝔲}\left(u\right)\right] \end{array}\right\}$, where 0 ≤ *u*_*α*_^*𝔲*^(*u*)+*v*_*α*_^*𝔲*^(*u*)+*w*_*α*_^*𝔲*^(*u*) ≤ 3 and *α*=1,2,3,…, *m*.

**Definition 9 .**

Let *℘*_*ℜ*_ and *℘*_*ℒ*_ be two mPIVNSSs over *𝒰*. Then, *℘*_*ℜ*_ is called an m-polar interval-valued neutrosophic soft subset of *℘*_*ℒ*_, if

(8) $$\begin{array}{c} u_{\alpha }^{\ell ℜ}\left(u\right)\geq u_{\alpha }^{\ell L}\left(u\right),\\ u_{\alpha }^{uℜ}\left(u\right)\geq u_{\alpha }^{uL}\left(u\right),\\ v_{\alpha }^{\ell ℜ}\left(u\right)\geq v_{\alpha }^{\ell L}\left(u\right),\\ v_{\alpha }^{uℜ}\left(u\right)\geq v_{\alpha }^{uL}\left(u\right),\\ w_{\alpha }^{\ell ℜ}\left(u\right)\geq w_{\alpha }^{\ell L}\left(u\right),\\ w_{\alpha }^{uℜ}\left(u\right)\geq w_{\alpha }^{uL}\left(u\right), \end{array}$$

for all *α*=1,2,3,…, *m*; *e* ∈ *ℰ* and *u* ∈ *𝒰*.

**Definition 10 .**

Let *℘*_*ℜ*_ and *℘*_*ℒ*_ be two mPIVNSSs over *𝒰*. Then, *℘*_*ℜ*_=*℘*_*ℒ*_, if

(9) $$\begin{array}{c} u_{\alpha }^{\ell ℜ}\left(u\right)\leq u_{\alpha }^{\ell L}\left(u\right),\\ u_{\alpha }^{\ell L}\left(u\right)\leq u_{\alpha }^{\ell ℜ}\left(u\right),\\ u_{\alpha }^{uℜ}\left(u\right)\leq u_{\alpha }^{uL}\left(u\right),\\ u_{\alpha }^{uL}\left(u\right)\leq u_{\alpha }^{uℜ}\left(u\right),\\ v_{\alpha }^{\ell ℜ}\left(u\right)\geq v_{\alpha }^{\ell L}\left(u\right),\\ v_{\alpha }^{\ell L}\left(u\right)\geq v_{\alpha }^{\ell ℜ}\left(u\right),\\ v_{\alpha }^{uℜ}\left(u\right)\geq v_{\alpha }^{uL}\left(u\right),\\ v_{\alpha }^{uL}\left(u\right)\geq v_{\alpha }^{uℜ}\left(u\right),\\ w_{\alpha }^{\ell ℜ}\left(u\right)\geq w_{\alpha }^{\ell L}\left(u\right),\\ w_{\alpha }^{\ell L}\left(u\right)\geq w_{\alpha }^{\ell ℜ}\left(u\right),\\ w_{\alpha }^{uℜ}\left(u\right)\geq w_{\alpha }^{uL}\left(u\right),\\ w_{\alpha }^{uL}\left(u\right)\geq w_{\alpha }^{uℜ}\left(u\right), \end{array}$$

for all *α*=1,2,3,…, *m*; *e* ∈ *ℰ* and *u* ∈ *𝒰*.

**Definition 11 .**

Let *℘*_*ℜ*_ be an mPIVNSS over *𝒰*. Then, empty mPIVNSS can be represented as ${℘}_{\overset{ˇ}{0}}$ and defined as follows:

(10) $$\begin{array}{c} {℘}_{\overset{ˇ}{0}}=\left\{\left(e,\left\{\left(u,\left(\left[0,0\right],\left[1,1\right],\left[1,1\right]\right),\left(\left[0,0\right],\left[1,1\right],\left[1,1\right]\right),\ldots ,\;\left(\left[0,0\right],\left[1,1\right],\left[1,1\right]\right)\right):u\in U\right\}\right):e\in E\right\}. \end{array}$$

**Definition 12 .**

Let *℘*_*ℜ*_ be an mPIVNSS over *𝒰*. Then, universal mPIVNSS can be represented as ${℘}_{\overset{ˇ}{\bar{E}}}$ and defined as follows:

(11) $$\begin{array}{c} {℘}_{\overset{ˇ}{\bar{E}}}=\left\{\left(e,\left\{\left(u,\left(\left[1,1\right],\left[0,0\right],\left[0,0\right]\right),\left(\left[1,1\right],\left[0,0\right],\left[0,0\right]\right),\ldots ,\;\left(\left[1,1\right],\left[0,0\right],\left[0,0\right]\right)\right):u\in U\right\}\right):e\in E\right\}. \end{array}$$

**Definition 13 .**

Let *℘*_*ℜ*_ be an mPIVNSS over *𝒰*. Then, the complement of mPIVNSS is defined as follows:

(12) $$\begin{array}{c} {℘}_{ℜ}^{c}=\left\{\left(e,\left\{\left(\begin{array}{c} u,\left[w_{\alpha }^{\ell }\left(u\right),w_{\alpha }^{u}\left(u\right)\right],\left[1-\;u_{\alpha }^{u}\left(u\right),1-\;u_{\alpha }^{\ell }\left(u\right)\right], \end{array}\left[v_{\alpha }^{\ell }\left(u\right),v_{\alpha }^{u}\left(u\right)\right]\right):u\in U,\alpha =1,\;2,\;3,\ldots ,\;m\right\}\right):e\in E\right\}. \end{array}$$

**Proposition 1 .**

If *℘*_*ℜ*_ be an mPIVNSS over *𝒰*, then

(13) $$\begin{array}{c} {\left({℘}_{ℜ}^{c}\right)}^{c}={℘}_{ℜ},\\ {\left({℘}_{\overset{ˇ}{0}}\right)}^{c}={℘}_{\overset{ˇ}{\bar{E}}},\\ {\left({℘}_{\overset{ˇ}{\bar{E}}}\right)}^{c}={℘}_{\overset{ˇ}{0}}. \end{array}$$

**Proof**

Let

(14) $$\begin{array}{c} {℘}_{ℜ}=\left\{\left(e,\left\{\left(u,\left[u_{\alpha }^{\ell }\left(u\right),u_{\alpha }^{u}\left(u\right)\right],\left[v_{\alpha }^{\ell }\left(u\right),v_{\alpha }^{u}\left(u\right)\right],\left[w_{\alpha }^{\ell }\left(u\right),w_{\alpha }^{u}\left(u\right)\right]\right):u\in U,\alpha =1,\;2,\;3,\ldots ,\;m\right\}\right):e\in E\right\}. \end{array}$$

Then, by using Definition 13, we get

(15) $$\begin{array}{c} {℘}_{ℜ}^{c}=\left\{\left(e,\left\{\left(\begin{array}{c} u,\left[w_{\alpha }^{\ell }\left(u\right),w_{\alpha }^{u}\left(u\right)\right],\left[1-v_{\alpha }^{u}\left(u\right),1-\;v_{\alpha }^{\ell }\left(u\right)\right], \end{array}\left[u_{\alpha }^{\ell }\left(u\right),u_{\alpha }^{u}\left(u\right)\right]\right):u\in U,\alpha =1,\;2,\;3,\ldots ,\;m\right\}\right):e\in E\right\}, \end{array}$$

again, by using Definition 13,

(16) $$\begin{array}{c} {\left({℘}_{ℜ}^{c}\right)}^{c}=\left\{\left(e,\left\{\left(u,\left[u_{\alpha }^{\ell }\left(u\right),u_{\alpha }^{u}\left(u\right)\right],\left[1-1+v_{\alpha }^{\ell }\left(u\right),1-1+v_{\alpha }^{u}\left(u\right)\right],\left[w_{\alpha }^{\ell }\left(u\right),w_{\alpha }^{u}\left(u\right)\right]\right):u\in U,\alpha =1,\;2,\;3,\ldots ,\;m\right\}\right):e\in E\right\},\\ {\left({℘}_{ℜ}^{c}\right)}^{c}=\left\{\left(e,\left\{\left(u,\left[u_{\alpha }^{\ell }\left(u\right),u_{\alpha }^{u}\left(u\right)\right],\left[v_{\alpha }^{\ell }\left(u\right),v_{\alpha }^{u}\left(u\right)\right],\left[w_{\alpha }^{\ell }\left(u\right),w_{\alpha }^{u}\left(u\right)\right]\right):u\in U,\alpha =1,\;2,\;3,\ldots ,\;m\right\}\right):e\in E\right\},\\ {\left({℘}_{ℜ}^{c}\right)}^{c}={℘}_{R}. \end{array}$$

Similarly, we can prove 2 and 3.

**Definition 14 .**

Let *℘*_*ℜ*_ and *℘*_*ℒ*_ be two mPIVNSSs over *𝒰*. Then,

(17) $$\begin{array}{c} {℘}_{ℜ}\cup {℘}_{L}=\left\{\begin{array}{c} \left(e,\left\{\left(\begin{array}{c} u,\left[\max\left\{u_{\alpha }^{\ell ℜ}\left(u\right),u_{\alpha }^{\ell L}\left(u\right)\right\},\max\left\{u_{\alpha }^{uℜ}\left(u\right),u_{\alpha }^{uL}\left(u\right)\right\}\right],\\ \left[\min\left\{v_{\alpha }^{\ell ℜ}\left(u\right),v_{\alpha }^{\ell L}\left(u\right)\right\},\min\left\{v_{\alpha }^{uℜ}\left(u\right),v_{\alpha }^{uL}\left(u\right)\right\}\right],\\ \left[\min\left\{w_{\alpha }^{\ell ℜ}\left(u\right),w_{\alpha }^{\ell L}\left(u\right)\right\},\min\left\{w_{\alpha }^{uℜ}\left(u\right),w_{\alpha }^{uL}\left(u\right)\right\}\right] \end{array}\right):u\in U,\alpha =1,2,3,\ldots ,m\right\}\right):e\in E \end{array}\right\}. \end{array}$$

**Definition 15 .**

Let *℘*_*ℜ*_ and *℘*_*ℒ*_ be two mPIVNSSs over *𝒰*. Then,

(18) $$\begin{array}{c} {℘}_{ℜ}\cap {℘}_{L}=\left\{\begin{array}{c} \left(e,\left\{\left(\begin{array}{c} u,\left[\min\left\{u_{\alpha }^{\ell ℜ}\left(u\right),u_{\alpha }^{\ell L}\left(u\right)\right\},\min\left\{u_{\alpha }^{uℜ}\left(u\right),u_{\alpha }^{uL}\left(u\right)\right\}\right],\\ \left[\max\left\{v_{\alpha }^{\ell ℜ}\left(u\right),v_{\alpha }^{\ell L}\left(u\right)\right\},\max\left\{v_{\alpha }^{uℜ}\left(u\right),v_{\alpha }^{uL}\left(u\right)\right\}\right],\\ \left[\max\left\{w_{\alpha }^{\ell ℜ}\left(u\right),w_{\alpha }^{\ell L}\left(u\right)\right\},\max\left\{w_{\alpha }^{uℜ}\left(u\right),w_{\alpha }^{uL}\left(u\right)\right\}\right] \end{array}\right):u\in U,\alpha =1,2,3,\ldots ,m\right\}\right):e\in E \end{array}\right\}. \end{array}$$

**Proposition 2 .**

Let *℘*_*ℜ*_ and *℘*_*ℒ*_ be two mPIVNSSs over *𝒰*. Then,

(19) $$\begin{array}{c} {\left({℘}_{ℜ}\cup {℘}_{L}\right)}^{C}={℘}_{ℜ}^{C}\cap {℘}_{L}^{C},\\ {\left({℘}_{ℜ}\cap {℘}_{L}\right)}^{C}={℘}_{ℜ}^{C}\cup {℘}_{L}^{C}. \end{array}$$

**Proof**

As we know,

(20) $$\begin{array}{c} {℘}_{R}=\left\{\begin{array}{c} \left(e,\left\{\left(u,\left[u_{\alpha }^{\ell R}\left(u\right),u_{\alpha }^{uR}\left(u\right)\right],\left[v_{\alpha }^{\ell R}\left(u\right),v_{\alpha }^{uR}\left(u\right)\right],\left[w_{\alpha }^{\ell R}\left(u\right),w_{\alpha }^{uR}\left(u\right)\right]\right):u\in U,\alpha =1,\;2,\;3,\ldots ,\;m\right\}\right):e\in E \end{array}\right\},\\ {℘}_{L}=\left\{\begin{array}{c} \left(e,\left\{\left(u,\left[u_{\alpha }^{\ell L}\left(u\right),u_{\alpha }^{uL}\left(u\right)\right],\left[v_{\alpha }^{\ell L}\left(u\right),v_{\alpha }^{uL}\left(u\right)\right],\left[w_{\alpha }^{\ell L}\left(u\right),w_{\alpha }^{uL}\left(u\right)\right]\right):u\in U,\alpha =1,\;2,\;3,\ldots ,\;m\right\}\right):e\in E \end{array}\right\}. \end{array}$$

By using Definition 14, we get

(21) $$\begin{array}{c} {℘}_{ℜ}\cup {℘}_{L}=\left\{\begin{array}{c} \left(e,\left\{\left(\begin{array}{c} u,\left[\max\left\{u_{\alpha }^{\ell ℜ}\left(u\right),\;u_{\alpha }^{\ell L}\left(u\right)\right\},\max\left\{u_{\alpha }^{uℜ}\left(u\right),\;u_{\alpha }^{uL}\left(u\right)\right\}\right],\\ \left[\min\left\{v_{\alpha }^{\ell ℜ}\left(u\right),\;v_{\alpha }^{\ell L}\left(u\right)\right\},\min\left\{v_{\alpha }^{uℜ}\left(u\right),\;v_{\alpha }^{uL}\left(u\right)\right\}\right],\\ \left[\min\left\{w_{\alpha }^{\ell ℜ}\left(u\right),\;w_{\alpha }^{\ell L}\left(u\right)\right\},\min\left\{w_{\alpha }^{uℜ}\left(u\right),\;w_{\alpha }^{uL}\left(u\right)\right\}\right] \end{array}\right):u\in U,\alpha =1,2,3,\ldots ,m\right\}\right):e\in E \end{array}\right\}. \end{array}$$

Now, by using Definition 13, we get

(22) $$\begin{array}{c} {\left({℘}_{ℜ}\cup {℘}_{L}\right)}^{c}=\left\{\begin{array}{c} \left(e,\left\{\left(\begin{array}{c} u,\left[\min\left\{w_{\alpha }^{\ell ℜ}\left(u\right),\;w_{\alpha }^{\ell L}\left(u\right)\right\},\min\left\{w_{\alpha }^{uℜ}\left(u\right),\;w_{\alpha }^{uL}\left(u\right)\right\}\right],\\ \left[1-\min\left\{v_{\alpha }^{uR}\left(u\right),\;v_{\alpha }^{uL}\left(u\right)\right\},1-\min\left\{v_{\alpha }^{\ell R}\left(u\right),\;v_{\alpha }^{\ell L}\left(u\right)\right\}\right],\\ \left[\max\left\{u_{\alpha }^{\ell ℜ}\left(u\right),\;u_{\alpha }^{\ell L}\left(u\right)\right\},\max\left\{u_{\alpha }^{uℜ}\left(u\right),\;u_{\alpha }^{uL}\left(u\right)\right\}\right] \end{array}\right):u\in U,\alpha =1,2,3,\ldots ,m\right\}\right):e\in E \end{array}\right\}. \end{array}$$

Now,

(23) $$\begin{array}{c} {℘}_{R}^{\text{C}}=\left\{\begin{array}{c} \left(e,\left\{\left(u,\left[w_{\alpha }^{\ell R}\left(u\right),w_{\alpha }^{uR}\left(u\right)\right],\left[1-v_{\alpha }^{\ell R}\left(u\right),1-v_{\alpha }^{uR}\left(u\right)\right],\left[u_{\alpha }^{\ell R}\left(u\right),u_{\alpha }^{uR}\left(u\right)\right]\right):u\in U,\alpha =1,\;2,\;3,\ldots ,\;m\right\}\right):e\in E \end{array}\right\},\\ {℘}_{L}^{\text{C}}=\left\{\begin{array}{c} \left(e,\left\{\left(u,\left[w_{\alpha }^{\ell L}\left(u\right),w_{\alpha }^{uL}\left(u\right)\right],\left[1-v_{\alpha }^{\ell L}\left(u\right),1-v_{\alpha }^{uL}\left(u\right)\right],\left[u_{\alpha }^{\ell L}\left(u\right),u_{\alpha }^{uL}\left(u\right)\right]\right):u\in U,\alpha =1,\;2,\;3,\ldots ,\;m\right\}\right):e\in E \end{array}\right\}. \end{array}$$

By using Definition 15,

(24) $$\begin{array}{c} {℘}_{R}^{C}\cap {℘}_{L}^{C}=\left\{\begin{array}{c} \left(e,\left\{\left(\begin{array}{c} u,\left[\min\left\{w_{\alpha }^{\ell ℜ}\left(u\right),\;w_{\alpha }^{\ell L}\left(u\right)\right\},\min\left\{w_{\alpha }^{uℜ}\left(u\right),\;w_{\alpha }^{uL}\left(u\right)\right\}\right],\\ \left[\min\left\{1-v_{\alpha }^{uR}\left(u\right),\;1-v_{\alpha }^{uL}\left(u\right)\right\},\min\left\{1-v_{\alpha }^{\ell R}\left(u\right),\;1-v_{\alpha }^{\ell L}\left(u\right)\right\}\right],\\ \left[\max\left\{u_{\alpha }^{\ell ℜ}\left(u\right),\;u_{\alpha }^{\ell L}\left(u\right)\right\},\max\left\{u_{\alpha }^{uℜ}\left(u\right),\;u_{\alpha }^{uL}\left(u\right)\right\}\right] \end{array}\right):u\in U,\alpha =1,2,3,\ldots ,m\right\}\right):e\in E \end{array}\right\},\\ {℘}_{R}^{C}\cap {℘}_{L}^{C}=\left\{\begin{array}{c} \left(e,\left\{\left(\begin{array}{c} u,\left[\min\left\{w_{\alpha }^{\ell ℜ}\left(u\right),\;w_{\alpha }^{\ell L}\left(u\right)\right\},\min\left\{w_{\alpha }^{uℜ}\left(u\right),\;w_{\alpha }^{uL}\left(u\right)\right\}\right],\\ \left[1-\min\left\{v_{\alpha }^{uR}\left(u\right),\;v_{\alpha }^{uL}\left(u\right)\right\},1-\min\left\{v_{\alpha }^{\ell R}\left(u\right),\;v_{\alpha }^{\ell L}\left(u\right)\right\}\right],\\ \left[\max\left\{u_{\alpha }^{\ell ℜ}\left(u\right),\;u_{\alpha }^{\ell L}\left(u\right)\right\},\max\left\{u_{\alpha }^{uℜ}\left(u\right),\;u_{\alpha }^{uL}\left(u\right)\right\}\right] \end{array}\right):u\in U,\alpha =1,2,3,\ldots ,m\right\}\right):e\in E \end{array}\right\}. \end{array}$$

Hence,

(25) $$\begin{array}{c} {\left({℘}_{ℜ}\cup {℘}_{L}\right)}^{c}={℘}_{ℜ}^{C}\cap {℘}_{L}^{C}. \end{array}$$

**Proof**

Similar to assertion 1.

**Proposition 3 .**

Let *℘*_*ℜ*_, *℘*_*ℒ*_, and *℘*_*ℋ*_ be three mPIVNSSs over *𝒰*. Then,

(26) $$\begin{array}{c} {℘}_{R}\cup \left({℘}_{L}\cap {℘}_{H}\right)=\left({℘}_{R}\cup {℘}_{L}\right)\cap \left({℘}_{R}\cup {℘}_{H}\right),\\ {℘}_{R}\cap \left({℘}_{L}\cup {℘}_{H}\right)=\left({℘}_{R}\cap {℘}_{L}\right)\cup \left({℘}_{R}\cap {℘}_{H}\right),\\ {℘}_{R}\cup \left({℘}_{R}\cap {℘}_{L}\right)={℘}_{R},\\ {℘}_{R}\cap \left({℘}_{R}\cup {℘}_{L}\right)={℘}_{R}. \end{array}$$

**Proof**

As we know,

(27) $$\begin{array}{c} {℘}_{L}\cap {℘}_{H}=\left\{\begin{array}{c} \left(e,\left\{\left(\begin{array}{c} u,\left[\min\left\{u_{\alpha }^{\ell L}\left(u\right),u_{\alpha }^{\ell H}\left(u\right)\right\},\min\left\{u_{\alpha }^{uL}\left(u\right),u_{\alpha }^{uH}\left(u\right)\right\}\right],\\ \left[\max\left\{v_{\alpha }^{\ell L}\left(u\right),v_{\alpha }^{\ell H}\left(u\right)\right\},\max\left\{v_{\alpha }^{uL}\left(u\right),v_{\alpha }^{uH}\left(u\right)\right\}\right],\\ \left[\max\left\{w_{\alpha }^{\ell L}\left(u\right),w_{\alpha }^{\ell H}\left(u\right)\right\},\max\left\{w_{\alpha }^{uL}\left(u\right),w_{\alpha }^{uH}\left(u\right)\right\}\right] \end{array}\right):u\in U,\alpha =1,2,3,\ldots ,m\right\}\right):e\in E \end{array}\right\},\\ {℘}_{ℜ}\cup \left({℘}_{L}\cap {℘}_{H}\right)=\left\{\begin{array}{c} \left(e,\;\left\{\left(\begin{array}{c} u,\left[\max\left\{u_{\alpha }^{\ell ℜ}\left(u\right),\min\left\{\min\left\{u_{\alpha }^{\ell L}\left(u\right),u_{\alpha }^{\ell H}\left(u\right)\right\}\right\}\right\},\max\left\{u_{\alpha }^{uℜ}\left(u\right),\min\left\{u_{\alpha }^{uL}\left(u\right),u_{\alpha }^{uH}\left(u\right)\right\}\;\right\}\right],\\ \left[\min\left\{v_{\alpha }^{\ell ℜ}\left(u\right),\max\left\{v_{\alpha }^{\ell L}\left(u\right),v_{\alpha }^{\ell H}\left(u\right)\right\}\right\},\min\left\{v_{\alpha }^{uℜ}\left(u\right),\max\left\{v_{\alpha }^{uL}\left(u\right),v_{\alpha }^{uH}\left(u\right)\right\}\;\right\}\right],\\ \left[\min\left\{w_{\alpha }^{\ell ℜ}\left(u\right),\max\left\{w_{\alpha }^{\ell L}\left(u\right),w_{\alpha }^{\ell H}\left(u\right)\right\}\right\},\min\left\{w_{\alpha }^{uℜ}\left(u\right),\max\left\{w_{\alpha }^{uL}\left(u\right),w_{\alpha }^{uH}\left(u\right)\right\}\;\right\}\right] \end{array}\right):u\in U,\alpha =1,2,3,\ldots ,m\right\}\right):e\in E \end{array}\right\},\\ {℘}_{ℜ}\cap {℘}_{L}=\left\{\begin{array}{c} \left(e,\;\left\{\left(\begin{array}{c} u,\left[\min\left\{u_{\alpha }^{\ell ℜ}\left(u\right),u_{\alpha }^{\ell L}\left(u\right)\right\},\min\left\{u_{\alpha }^{uℜ}\left(u\right),u_{\alpha }^{uL}\left(u\right)\right\}\right],\\ \left[\max\left\{v_{\alpha }^{\ell ℜ}\left(u\right),v_{\alpha }^{\ell L}\left(u\right)\right\},\max\left\{v_{\alpha }^{uℜ}\left(u\right),v_{\alpha }^{uL}\left(u\right)\right\}\right],\\ \left[\max\left\{w_{\alpha }^{\ell ℜ}\left(u\right),w_{\alpha }^{\ell L}\left(u\right)\right\},\max\left\{w_{\alpha }^{uℜ}\left(u\right),w_{\alpha }^{uL}\left(u\right)\right\}\right] \end{array}\right):u\in U,\alpha =1,2,3,\ldots ,m\right\}\right):e\in E \end{array}\right\},\\ {℘}_{ℜ}\cap {℘}_{H}=\left\{\begin{array}{c} \left(e,\;\left\{\left(\begin{array}{c} u,\left[\min\left\{u_{\alpha }^{\ell ℜ}\left(u\right),u_{\alpha }^{\ell H}\left(u\right)\right\},\min\left\{u_{\alpha }^{uℜ}\left(u\right),u_{\alpha }^{uH}\left(u\right)\right\}\right],\\ \left[\max\left\{v_{\alpha }^{\ell ℜ}\left(u\right),v_{\alpha }^{\ell H}\left(u\right)\right\},\max\left\{v_{\alpha }^{uℜ}\left(u\right),v_{\alpha }^{uH}\left(u\right)\right\}\right],\\ \left[\max\left\{w_{\alpha }^{\ell ℜ}\left(u\right),w_{\alpha }^{\ell H}\left(u\right)\right\},\max\left\{w_{\alpha }^{uℜ}\left(u\right),w_{\alpha }^{uH}\left(u\right)\right\}\right] \end{array}\right):u\in U,\alpha =1,2,3,\ldots ,m\right\}\right):e\in E \end{array}\right\},\\ \left({℘}_{ℜ}\cap {℘}_{L}\right)\cup \left({℘}_{ℜ}\cap {℘}_{H}\right)=\left\{\begin{array}{c} \left(e,\left\{\left(\begin{array}{c} u,\left[\begin{array}{c} \max\left\{\min\left\{u_{\alpha }^{\ell ℜ}\left(u\right),u_{\alpha }^{\ell L}\left(u\right)\right\},\min\left\{u_{\alpha }^{\ell ℜ}\left(u\right),u_{\alpha }^{\ell H}\left(u\right)\right\}\right\},\max\left\{\min\left\{u_{\alpha }^{uℜ}\left(u\right),u_{\alpha }^{uL}\left(u\right)\right\},\min\left\{u_{\alpha }^{uℜ}\left(u\right),u_{\alpha }^{uH}\left(u\right)\right\}\;\right\} \end{array}\right],\\ \left[\begin{array}{c} \min\left\{\max\left\{v_{\alpha }^{\ell ℜ}\left(u\right),v_{\alpha }^{\ell L}\left(u\right)\right\},\max\left\{v_{\alpha }^{\ell ℜ}\left(u\right),v_{\alpha }^{\ell H}\left(u\right)\right\}\right\},\min\left\{\max\left\{v_{\alpha }^{uℜ}\left(u\right),v_{\alpha }^{uL}\left(u\right)\right\},\max\left\{v_{\alpha }^{uℜ}\left(u\right),v_{\alpha }^{uH}\left(u\right)\right\}\right\} \end{array}\right],\\ \left[\begin{array}{c} \min\left\{\max\left\{w_{\alpha }^{\ell ℜ}\left(u\right),w_{\alpha }^{\ell L}\left(u\right)\right\},\max\left\{w_{\alpha }^{\ell ℜ}\left(u\right),w_{\alpha }^{\ell H}\left(u\right)\right\}\right\},\min\left\{\max\left\{w_{\alpha }^{uℜ}\left(u\right),w_{\alpha }^{uL}\left(u\right)\right\},\max\left\{w_{\alpha }^{uℜ}\left(u\right),w_{\alpha }^{uH}\left(u\right)\right\}\right\} \end{array}\right] \end{array}\right):u\in U,\;\alpha =1,\;2,\;3,\ldots ,\;m\right\}\right):e\in E \end{array}\right\},\\ \left({℘}_{ℜ}\cap {℘}_{L}\right)\cup \left({℘}_{ℜ}\cap {℘}_{H}\right)=\left\{\begin{array}{c} \left(e,\;\left\{\left(\begin{array}{c} u,\left[\max\left\{u_{\alpha }^{\ell ℜ}\left(u\right),\min\left\{u_{\alpha }^{\ell L}\left(u\right),u_{\alpha }^{\ell H}\left(u\right)\right\}\right\},\max\left\{u_{\alpha }^{uℜ}\left(u\right),\min\left\{u_{\alpha }^{uL}\left(u\right),u_{\alpha }^{uH}\left(u\right)\right\}\right\}\right],\\ \left[\min\left\{v_{\alpha }^{\ell ℜ}\left(u\right),\max\left\{v_{\alpha }^{\ell L}\left(u\right),v_{\alpha }^{\ell H}\left(u\right)\right\}\right\},\min\left\{v_{\alpha }^{uℜ}\left(u\right),\max\left\{v_{\alpha }^{uL}\left(u\right),v_{\alpha }^{uH}\left(u\right)\right\}\right\}\right],\\ \left[\min\left\{w_{\alpha }^{\ell ℜ}\left(u\right),\max\left\{w_{\alpha }^{\ell L}\left(u\right),w_{\alpha }^{\ell H}\left(u\right)\right\}\right\},\min\left\{w_{\alpha }^{uℜ}\left(u\right),\max\left\{w_{\alpha }^{uL}\left(u\right),w_{\alpha }^{uH}\left(u\right)\right\}\right\}\right] \end{array}\right):u\in U,\;\alpha =1,\;2,\;3,\ldots ,\;m\right\}\right):e\in E \end{array}\right\}. \end{array}$$

Hence,

(28) $$\begin{array}{c} {℘}_{ℜ}\cup \left({℘}_{L}\cap {℘}_{H}\right)=\left({℘}_{ℜ}\cap {℘}_{L}\right)\cup \left({℘}_{ℜ}\cap {℘}_{H}\right). \end{array}$$

Similarly, we can prove other results.

**Definition 16 .**

Let *℘*_*R*_ and *℘*_*ℒ*_ be two mPIVNSSs over *𝒰*. Then, their extended union is defined as

(29) $$\begin{array}{c} u\left({℘}_{R}\cup_{\epsilon }{℘}_{L}\right)=\left\{\begin{array}{cc} \left[u_{\alpha }^{\ell R}\left(u\right),\;u_{\alpha }^{uR}\left(u\right)\right], & \text{if }e\in R-L,\\ \left[u_{\alpha }^{\ell L}\left(u\right),\;u_{\alpha }^{uL}\left(u\right)\right], & \text{if }e\in L-ℜ,\\ \left[\max\left\{u_{\alpha }^{\ell R}\left(u\right),u_{\alpha }^{\ell L}\left(u\right)\right\},\text{max}\left\{u_{\alpha }^{uR}\left(u\right),u_{\alpha }^{uL}\left(u\right)\right\}\right], & \text{if }e\in ℜ\cap L, \end{array}\right.\\ v\left({℘}_{R}\cup_{\epsilon }{℘}_{L}\right)=\left\{\begin{array}{cc} \left[v_{\alpha }^{\ell R}\left(u\right),\;v_{\alpha }^{uR}\left(u\right)\right], & \text{if }e\in ℜ-L,\\ \left[v_{\alpha }^{\ell L}\left(u\right),\;v_{\alpha }^{uL}\left(u\right)\right], & \text{if }e\in L-ℜ,\\ \left[\min\left\{v_{\alpha }^{\ell R}\left(u\right),v_{\alpha }^{\ell L}\left(u\right)\right\},\text{min}\left\{v_{\alpha }^{uR}\left(u\right),v_{\alpha }^{uL}\left(u\right)\right\}\right], & \text{if }e\in ℜ\cap L, \end{array}\right.\\ w\left({℘}_{R}\cup_{\epsilon }{℘}_{L}\right)=\left\{\begin{array}{cc} \left[w_{\alpha }^{\ell R}\left(u\right),w_{\alpha }^{uR}\left(u\right)\right], & \text{if }e\in ℜ-L,\\ \left[w_{\alpha }^{\ell L}\left(u\right),w_{\alpha }^{uL}\left(u\right)\right], & \text{if }e\in L-ℜ,\\ \left[\min\left\{w_{\alpha }^{\ell R}\left(u\right),w_{\alpha }^{\ell L}\left(u\right)\right\},\text{min}\left\{w_{\alpha }^{uR}\left(u\right),w_{\alpha }^{uL}\left(u\right)\right\}\right], & \text{if }e\in ℜ\cap L. \end{array}\right. \end{array}$$

**Example 1 .**

Assume *𝒰*={*u*_1_, *u*_2_} be a universe of discourse and *E*={*e*_1_, *e*_2_, *e*_3_, *e*_4_} be a set of attributes, and *ℜ*={*e*_1_, *e*_2_} and *ℒ*=Unsupported{*e*_3_, *e*_4_}⊆*E*. Consider 3-PIVNSSs *℘*_*ℜ*_ and *℘*_*ℒ*_ over *𝒰* can be represented as follows:

(30) $$\begin{array}{c} {℘}_{ℜ}=\left\{\begin{array}{c} \left(\begin{array}{c} e_{1},\;\left\{\begin{array}{c} \left(u_{1},\left(\left[0.5,0.8\right],\;\left[0.2,0.5\right],\;\left[0.1,0.2\right]\right),\left(\left[0.3,0.5\right],\left[0.1,0.3\right],\;\left[0.2,0.4\right]\right),\left(\left[0.6,0.9\right],\left[0.7,0.8\right],\left[0.8,1\right]\right)\right)\\ \left(u_{2},\left(\left[0.2,0.4\right],\left[0.3,0.4\right],\left[0.1,0.3\right]\right),\left(\left[0.2,0.5\right],\left[0.1,0.6\right],\left[0.1,0.3\right]\right),\left(\left[0.8,\;1\right],\left[0.6,0.9\right],\left[0.6,0.7\right]\right)\right) \end{array}\right\} \end{array}\right),\\ \left(\begin{array}{c} e_{2},\;\left\{\begin{array}{c} \left(u_{1},\left(\left[0.3,0.6\right],\left[0.1,0.6\right],\left[0.3,0.4\right]\right),\left(\left[0,0.2\right],\left[0.1,0.4\right],\left[0.3,0.5\right]\right),\left(\left[0.5,0.9\right],\left[0.3,0.8\right],\left[0.5,0.8\right]\right)\right)\\ \left(u_{2},\left(\left[0.2,0.5\right],\left[0.2,0.3\right],\left[0.5,0.6\right]\right),\left(\left[0.3,0.5\right],\left[0.1,0.5\right],\left[0.5,0.8\right]\right),\left(\left[0.6,0.9\right],\left[0.5,0.8\right],\left[0.6,0.9\right]\right)\right) \end{array}\right\} \end{array}\right) \end{array}\right\},\\ {℘}_{L}=\left\{\begin{array}{c} \left(\begin{array}{c} e_{1},\;\left\{\begin{array}{c} \left(u_{1},\left(\left[0.4,0.8\right],\left[0.3,0.6\right],\left[0.2,0.5\right]\right),\left(\left[0.2,0.7\right],\left[0.3,0.4\right],\left[0.4,0.6\right]\right),\left(\left[0.7,0.8\right],\left[0.4,0.9\right],\left[0.5,\;1\right]\right)\right)\\ \left(u_{2},\left(\left[0.1,0.6\right],\left[0.5,0.7\right],\;\left[0.1,0.2\right]\right),\left(\left[0.3,0.4\right],\;\left[0.2,0.5\right],\left[0.2,0.5\right]\right),\left(\left[0.5,0.9\right],\left[0.7,0.8\right],\left[0.4,0.6\right]\right)\right) \end{array}\right\} \end{array}\right),\\ \left(\begin{array}{c} e_{2},\;\left\{\begin{array}{c} \left(u_{1},\left(\left[0.2,0.7\right],\left[0.3,0.5\right],\left[0.2,0.6\right]\right),\left(\left[0.1,0.3\right],\left[0.2,0.5\right],\left[0.2,0.7\right]\right),\left(\left[0.4,0.9\right],\left[0.4,0.7\right],\left[0.5,0.8\right]\right)\right)\\ \left(u_{2},\left(\left[0.1,0.6\right],\left[0.1,0.5\right],\left[0.4,0.8\right]\right),\left(\left[0.3,0.6\right],\left[0.3,0.4\right],\left[1,1\right]\right),\left(\left[0.5,0.9\right],\left[0.3,0.7\right],\left[0.1,0.8\right]\right)\right) \end{array}\right\} \end{array}\right) \end{array}\right\}. \end{array}$$

Then,

(31) $$\begin{array}{c} {℘}_{R}\cup_{\epsilon }{℘}_{L}=\left\{\begin{array}{c} \left(\begin{array}{c} {\text{e}}_{1},\;\left\{\begin{array}{c} \left(u_{1},\left(\left[0.5,0.8\right],\;\left[0.2,0.5\right],\;\left[0.1,0.2\right]\right),\left(\left[0.3,0.5\right],\left[0.1,0.3\right],\;\left[0.2,0.4\right]\right),\left(\left[0.6,0.9\right],\left[0.7,0.8\right],\left[0.8,1\right]\right)\right)\\ \left(u_{2},\left(\left[0.2,0.4\right],\left[0.3,0.4\right],\left[0.1,0.3\right]\right),\left(\left[0.2,0.5\right],\left[0.1,0.6\right],\left[0.1,0.3\right]\right),\left(\left[0.8,\;1\right],\left[0.6,0.9\right],\left[0.6,0.7\right]\right)\right) \end{array}\right\} \end{array}\right),\\ \begin{array}{c} \left(\begin{array}{c} {\text{e}}_{2},\;\left\{\begin{array}{c} \left(u_{1},\left(\left[0.4,0.8\right],\left[0.1,0.6\right],\left[0.2,0.4\right]\right),\left(\left[0.2,0.7\right],\left[0.1,0.4\right],\left[0.3,0.5\right]\right),\;\left(\left[0.7,0.9\right],\left[0.3,0.8\right],\left[0.5,0.8\right]\right)\right)\\ \left(u_{2},\left(\left[0.2,0.6\right],\left[0.2,0.3\right],\left[0.1,0.2\right]\right),\left(\left[0.3,0.5\right],\left[0.1,0.5\right],\left[0.2,0.5\right]\right),\;\left(\left[0.6,0.9\right],\;\left[0.5,0.8\right],\left[.4,0.6\right]\right)\right) \end{array}\right\} \end{array}\right)\\ \left(\begin{array}{c} {\text{e}}_{2},\;\left\{\begin{array}{c} \left(u_{1},\left(\left[0.2,0.7\right],\left[0.3,0.5\right],\left[0.2,0.6\right]\right),\left(\left[0.1,0.3\right],\left[0.2,0.5\right],\left[0.2,0.7\right]\right),\left(\left[0.4,0.9\right],\left[0.4,0.7\right],\left[0.5,0.8\right]\right)\right)\\ \left(u_{2},\left(\left[0.1,0.6\right],\left[0.1,0.5\right],\left[0.4,0.8\right]\right),\left(\left[0.3,0.6\right],\left[0.3,0.4\right],\left[1,1\right]\right),\left(\left[0.5,0.9\right],\left[0.3,0.7\right],\left[0.1,0.8\right]\right)\right) \end{array}\right\} \end{array}\right) \end{array} \end{array}\right\}. \end{array}$$

**Definition 17 .**

Let *℘*_*R*_ and *℘*_*ℒ*_ be two mPIVNSSs over *𝒰*. Then, their extended intersection is defined as

(32) $$\begin{array}{c} u\left({℘}_{R}\cap_{\epsilon }{℘}_{L}\right)=\left\{\begin{array}{cc} \left[u_{\alpha }^{\ell R}\left(u\right),\;u_{\alpha }^{uR}\left(u\right)\right], & \text{if }e\in ℜ-L,\\ \left[u_{\alpha }^{\ell L}\left(u\right),\;u_{\alpha }^{uL}\left(u\right)\right], & \text{if }e\in L-ℜ,\\ \left[\min\left\{u_{\alpha }^{\ell R}\left(u\right),u_{\alpha }^{\ell L}\left(u\right)\right\},\text{min}\left\{u_{\alpha }^{uR}\left(u\right),u_{\alpha }^{uL}\left(u\right)\right\}\right], & \text{if }e\in ℜ\cap L, \end{array}\right.\\ v\left({℘}_{R}\cap_{\epsilon }{℘}_{L}\right)=\left\{\begin{array}{cc} \left[v_{\alpha }^{\ell R}\left(u\right),\;v_{\alpha }^{uR}\left(u\right)\right], & \text{if }e\in ℜ-L,\\ \left[v_{\alpha }^{\ell L}\left(u\right),\;v_{\alpha }^{uL}\left(u\right)\right], & \text{if }e\in L-ℜ,\\ \left[\max\left\{v_{\alpha }^{\ell R}\left(u\right),v_{\alpha }^{\ell L}\left(u\right)\right\},\text{max}\left\{v_{\alpha }^{uR}\left(u\right),v_{\alpha }^{uL}\left(u\right)\right\}\right], & \text{if }e\in ℜ\cap L, \end{array}\right.\\ w\left({℘}_{R}\cap_{\epsilon }{℘}_{L}\right)=\left\{\begin{array}{cc} \left[w_{\alpha }^{\ell R}\left(u\right),w_{\alpha }^{uR}\left(u\right)\right], & \text{if }e\in ℜ-L,\\ \left[w_{\alpha }^{\ell L}\left(u\right),w_{\alpha }^{uL}\left(u\right)\right], & \text{if }e\in L-ℜ,\\ \left[\max\left\{w_{\alpha }^{\ell R}\left(u\right),w_{\alpha }^{\ell L}\left(u\right)\right\},\max\left\{w_{\alpha }^{uR}\left(u\right),w_{\alpha }^{uL}\left(u\right)\right\}\right], & \text{if }e\in ℜ\cap L. \end{array}\right. \end{array}$$

**Remark 1 .**

Generally, if ${℘}_{ℜ}\neq {℘}_{\overset{ˇ}{0}}$ and ${℘}_{ℜ}\neq {℘}_{\overset{ˇ}{\bar{E}}}$, then the law of contradiction ${℘}_{ℜ}\cap {℘}_{ℜ}^{C}={℘}_{\overset{ˇ}{0}}$ and the law of the excluded middle ${℘}_{ℜ}\cup {℘}_{ℜ}^{C}={℘}_{\overset{ˇ}{\bar{E}}}$ do not satisfy mPIVNSS. On the other hand, in classical set theory, both laws always hold.

**Definition 18 .**

Let *℘*_*ℜ*_ and *℘*_*ℒ*_ be two mPIVNSSs over *𝒰*. Then, their difference is defined as follows:

(33) $$\begin{array}{c} {℘}_{ℜ}\backslash {℘}_{L}=\left\{\begin{array}{c} \left(e,\;\left\{\left(\begin{array}{c} u,\left[\min\left\{u_{\alpha }^{\ell ℜ}\left(u\right),u_{\alpha }^{\ell L}\left(u\right)\right\},\min\left\{u_{\alpha }^{uℜ}\left(u\right),u_{\alpha }^{uL}\left(u\right)\right\}\right],\\ \left[\max\left\{v_{\alpha }^{\ell ℜ}\left(u\right),1-v_{\alpha }^{uL}\left(u\right)\right\},\max\left\{v_{\alpha }^{uℜ}\left(u\right),1-v_{\alpha }^{\ell L}\left(u\right)\right\}\right],\\ \;\left[\max\left\{w_{\alpha }^{\ell ℜ}\left(u\right),w_{\alpha }^{\ell L}\left(u\right)\right\},\max\left\{w_{\alpha }^{uℜ}\left(u\right),w_{\alpha }^{uL}\left(u\right)\right\}\right] \end{array}\right):u\in U,\alpha =1,2,3,\ldots ,\;m\right\}\right):e\in E \end{array}\right\}. \end{array}$$

**Definition 19 .**

Let *℘*_*ℜ*_ and *℘*_*ℒ*_ be two mPIVNSSs over *𝒰*. Then, their addition is defined as follows:

(34) $$\begin{array}{c} {℘}_{ℜ}+{℘}_{L}=\left\{\begin{array}{c} \left(e,\left\{\left(\begin{array}{c} u,\left[\min\left\{u_{\alpha }^{\ell ℜ}\left(u\right)+\;u_{\alpha }^{\ell L}\left(u\right),\;1\right\},\min\left\{u_{\alpha }^{uℜ}\left(u\right)+u_{\alpha }^{uL}\left(u\right),\;1\right\}\right],\\ \left[\min\left\{v_{\alpha }^{\ell ℜ}\left(u\right)+v_{\alpha }^{\ell L}\left(u\right),\;1\right\},\min\left\{v_{\alpha }^{uℜ}\left(u\right)+v_{\alpha }^{uL}\left(u\right),\;1\right\}\right],\\ \;\left[\min\left\{w_{\alpha }^{\ell ℜ}\left(u\right)+\;w_{\alpha }^{\ell L}\left(u\right),\;1\right\},\min\left\{w_{\alpha }^{uℜ}\left(u\right)+w_{\alpha }^{uL}\left(u\right),\;1\right\}\right] \end{array}\right):u\in U,\alpha =1,2,3,\ldots ,m\right\}\right):e\in E \end{array}\right\}. \end{array}$$

**Definition 20 .**

Let *℘*_*ℜ*_ be an mPIVNSS over *𝒰*. Then, its scalar multiplication is represented as *℘*_*ℜ*_. $\overset{ˇ}{\text{a}}$, where $\overset{ˇ}{\text{a}}\in \left[0,1\right]$ and defined as follows:

(35) $$\begin{array}{c} {℘}_{ℜ}\cdot \overset{ˇ}{a}=\left\{\begin{array}{c} \left(e,\;\left\{\left(\begin{array}{c} u,\left[\min\left\{u_{\alpha }^{\ell ℜ}\left(u\right).\overset{ˇ}{a},\;1\right\},\min\left\{u_{\alpha }^{uℜ}\left(u\right).\overset{ˇ}{a},\;1\right\}\right],\\ \left[\min\left\{v_{\alpha }^{\ell ℜ}\left(u\right).\overset{ˇ}{a},\;1\right\},\min\left\{v_{\alpha }^{uℜ}\left(u\right).\overset{ˇ}{a},\;1\right\}\right],\\ \left[\min\left\{w_{\alpha }^{\ell ℜ}\left(u\right).\overset{ˇ}{a},\;1\right\},\min\left\{w_{\alpha }^{uℜ}\left(u\right).\overset{ˇ}{a},1\right\}\right] \end{array}\right):u\in U,\alpha =1,2,3,\ldots ,m\right\}\right):\;e\in E \end{array}\right\}. \end{array}$$

**Definition 21 .**

Let *℘*_*ℜ*_ be the mPIVNSS over *𝒰*. Then, its scalar division is represented as ${℘}_{ℜ}/\overset{ˇ}{a}$, where $\overset{ˇ}{\text{a}}\in \left[0,1\right]$ and defined as follows:

(36) $$\begin{array}{c} \frac{{℘}_{ℜ}}{\overset{ˇ}{a}}=\left\{\begin{array}{c} \left(e,\left\{\left(\begin{array}{c} u,\left[\min\left\{\frac{u_{\alpha }^{\ell ℜ}\left(u\right)}{\overset{ˇ}{a}},\;1\right\},\min\left\{\frac{u_{\alpha }^{uℜ}\left(u\right)}{\overset{ˇ}{a}},\;1\right\}\right],\\ \left[\min\left\{\frac{v_{\alpha }^{\ell ℜ}\left(u\right)}{\overset{ˇ}{a}},\;1\right\},\min\left\{\frac{v_{\alpha }^{uℜ}\left(u\right)}{\overset{ˇ}{a}},\;1\right\}\right],\\ \left[\min\left\{\frac{w_{\alpha }^{\ell ℜ}\left(u\right)}{\overset{ˇ}{a}},\;1\right\},\min\left\{\frac{w_{\alpha }^{uℜ}\left(u\right)}{\overset{ˇ}{a}},\;1\right\}\right] \end{array}\right):u\in U,\alpha =1,\;2,\;3,\ldots ,\;m\right\}\right):e\in E \end{array}\right\}. \end{array}$$

**Definition 22 .**

Let *℘*_*ℜ*_ be an mPIVNSS over *𝒰*. Then, the truth-favorite operator on *℘*_*ℜ*_ is denoted by $\tilde{\Delta }{℘}_{ℜ}$ and defined as follows:

(37) $$\begin{array}{c} \tilde{\Delta }{℘}_{ℜ}=\left\{\begin{array}{c} \left(e,\left\{\left(\begin{array}{c} u,\left[\min\left\{u_{\alpha }^{\ell ℜ}\left(u\right)+v_{\alpha }^{\ell ℜ}\left(u\right),1\right\},\min\left\{u_{\alpha }^{uℜ}\left(u\right)+v_{\alpha }^{uℜ}\left(u\right),1\right\}\right],\\ \left[0,0\right],\left[0,0\right],\ldots ,\;\left[0,0\right],\left[w_{\alpha }^{\ell ℜ}\left(u\right),w_{\alpha }^{uℜ}\left(u\right)\right] \end{array}\right):u\in U,\alpha =1,2,3,\ldots ,m\right\}\right):e\in E \end{array}\right\}. \end{array}$$

**Definition 23 .**

Let *℘*_*ℜ*_ be an mPIVNSS over *𝒰*. Then, the false-favorite operator on *℘*_*ℜ*_ is denoted by $\tilde{\Delta }{℘}_{ℜ}$ and is defined as follows:

(38) $$\begin{array}{c} \tilde{\nabla }{℘}_{ℜ}=\left\{\left(e,\left\{\left(\begin{array}{c} u,\left[u_{\alpha }^{\ell ℜ}\left(u\right),u_{\alpha }^{uℜ}\left(u\right)\right],\left[0,0\right],\left[0,0\right],\ldots ,\;\left[0,0\right],\\ \left[\min\left\{w_{\alpha }^{\ell ℜ}\left(u\right)+v_{\alpha }^{\ell ℜ}\left(u\right),1\right\},\min\left\{w_{\alpha }^{uℜ}\left(u\right)+v_{\alpha }^{uℜ}\left(u\right),1\right\}\right] \end{array}\right):u\in U,\alpha =1,2,3,\ldots ,m\right\}\right):e\in E\right\}. \end{array}$$

**Definition 24 .**

Let *℘*_*ℜ*_ and *℘*_*ℒ*_ be two mPIVNSSs over *𝒰*. Then, their AND operator is represented by *℘*_*ℜ*_∧*℘*_*ℒ*_ and defined as follows:

(39) $$\begin{array}{c} {℘}_{ℜ}\wedge {℘}_{L}=\neg_{ℜ\times L},\text{ where }\neg_{ℜ\times L}\left(x,\;y\right)={℘}_{ℜ}\left(x\right)\cap {℘}_{L}\left(y\right)\text{ for all }\left(x,\;y\right)\in ℜ\times L. \end{array}$$

**Definition 25 .**

Let *℘*_*ℜ*_ and *℘*_*ℒ*_ be two mPIVNSSs over *𝒰*. Then, their OR operator is represented by *℘*_*ℜ*_∨*℘*_*ℒ*_ and defined as follows:

(40) $$\begin{array}{c} {℘}_{ℜ}\vee {℘}_{L}=\neg_{ℜ\times L},\text{ where }\neg_{ℜ\times L}\left(x,\;y\right)={℘}_{ℜ}\left(x\right)\cup {℘}_{L}\left(y\right)\text{ for all }\left(x,\;y\right)\in ℜ\times L. \end{array}$$

**Example 2 .**

Reconsider Example 1.

(41) $$\begin{array}{c} {℘}_{ℜ}=\left\{\begin{array}{c} \left(\begin{array}{c} e_{1},\;\left\{\begin{array}{c} \left(u_{1},\left(\left[0.5,0.8\right],\;\left[0.2,0.5\right],\;\left[0.1,0.2\right]\right),\left(\left[0.3,0.5\right],\left[0.1,0.3\right],\;\left[0.2,0.4\right]\right),\left(\left[0.6,0.9\right],\left[0.7,0.8\right],\left[0.8,1\right]\right)\right)\\ \left(u_{2},\left(\left[0.2,0.4\right],\left[0.3,0.4\right],\left[0.1,0.3\right]\right),\left(\left[0.2,0.5\right],\left[0.1,0.6\right],\left[0.1,0.3\right]\right),\left(\left[0.8,\;1\right],\left[0.6,0.9\right],\left[0.6,0.7\right]\right)\right) \end{array}\right\} \end{array}\right),\\ \left(\begin{array}{c} e_{2},\;\left\{\begin{array}{c} \left(u_{1},\left(\left[0.3,0.6\right],\left[0.1,0.6\right],\left[0.3,0.4\right]\right),\left(\left[0,0.2\right],\left[0.1,0.4\right],\left[0.3,0.5\right]\right),\left(\left[0.5,0.9\right],\left[0.3,0.8\right],\left[0.5,0.8\right]\right)\right)\\ \left(u_{2},\left(\left[0.2,0.5\right],\left[0.2,0.3\right],\left[0.5,0.6\right]\right),\left(\left[0.3,0.5\right],\left[0.1,0.5\right],\left[0.5,0.8\right]\right),\left(\left[0.6,0.9\right],\left[0.5,0.8\right],\left[0.6,0.9\right]\right)\right) \end{array}\right\} \end{array}\right) \end{array}\right\},\\ {℘}_{L}=\left\{\begin{array}{c} \left(\begin{array}{c} e_{1},\;\left\{\begin{array}{c} \left(u_{1},\left(\left[0.4,0.8\right],\left[0.3,0.6\right],\left[0.2,0.5\right]\right),\left(\left[0.2,0.7\right],\left[0.3,0.4\right],\left[0.4,0.6\right]\right),\left(\left[0.7,0.8\right],\left[0.4,0.9\right],\left[0.5,\;1\right]\right)\right)\\ \left(u_{2},\left(\left[0.1,0.6\right],\left[0.5,0.7\right],\;\left[0.1,0.2\right]\right),\left(\left[0.3,0.4\right],\;\left[0.2,0.5\right],\left[0.2,0.5\right]\right),\left(\left[0.5,0.9\right],\left[0.7,0.8\right],\left[0.4,0.6\right]\right)\right) \end{array}\right\} \end{array}\right),\\ \left(\begin{array}{c} e_{2},\;\left\{\begin{array}{c} \left(u_{1},\left(\left[0.2,0.7\right],\left[0.3,0.5\right],\left[0.2,0.6\right]\right),\left(\left[0.1,0.3\right],\left[0.2,0.5\right],\left[0.2,0.7\right]\right),\left(\left[0.4,0.9\right],\left[0.4,0.7\right],\left[0.5,0.8\right]\right)\right)\\ \left(u_{2},\left(\left[0.1,0.6\right],\left[0.1,0.5\right],\left[0.4,0.8\right]\right),\left(\left[0.3,0.6\right],\left[0.3,0.4\right],\left[1,1\right]\right),\left(\left[0.5,0.9\right],\left[0.3,0.7\right],\left[0.1,0.8\right]\right)\right) \end{array}\right\} \end{array}\right) \end{array}\right\},\\ {℘}_{ℜ}\wedge {℘}_{L}=\left\{\begin{array}{c} \begin{array}{c} \left(e_{1},e_{2}\right),\left(u_{1},\left(\left[0.4,0.8\right],\left[0.3,0.6\right],\left[0.2,0.5\right]\right),\left(\left[0.2,0.5\right],\left[0.3,0.4\right],\left[0.4,0.6\right]\right),\left(\left[0.6,0.8\right],\left[0.7,0.9\right].\left[0.8,0.1\right]\right)\right),\\ \left(u_{2},\left(\left[0.1,0.4\right],\left[0.5,0.7\right],\left[0.1,0.3\right]\right),\left(\left[0.2,0.4\right],\left[0.2,0.6\right],\left[0.2,0.5\right]\right),\left(\left[0.5,0.9\right],\left[0.7,0.9\right].\left[0.6,0.7\right]\right)\right), \end{array}\\ \begin{array}{c} \left(e_{1},e_{3}\right),\left(u_{1},\left(\left[0.2,0.7\right],\left[0.3,0.5\right],\left[0.2,0.6\right]\right),\left(\left[0.1,0.3\right],\left[0.2,0.5\right],\left[0.2,0.7\right]\right),\left(\left[0.4,0.9\right],\left[0.7,0.8\right].\left[0.8,0.1\right]\right)\right),\\ \left(u_{2},\left(\left[0.1,0.4\right],\left[0.3,0.5\right],\left[0.4,0.8\right]\right),\left(\left[0.2,0.5\right],\left[0.3,0.6\right],\left[1,1\right]\right),\left(\left[0.5,0.9\right],\left[0.6,0.9\right].\left[0.6,0.8\right]\right)\right), \end{array}\\ \begin{array}{c} \left(e_{1},e_{2}\right),\left(u_{1},\left(\left[0.3,0.6\right],\left[0.1,0.6\right],\left[0.3,0.4\right]\right),\left(\left[0,0.2\right],\left[0.1,0.4\right],\left[0.3,0.5\right]\right),\left(\left[0.5,0.9\right],\left[0.3,0.8\right].\left[0.5,0.8\right]\right)\right),\\ \left(u_{2},\left(\left[0.2,0.5\right],\left[0.2,0.3\right],\left[0.5,0.6\right]\right),\left(\left[0.3,0.5\right],\left[0.1,0.5\right],\left[0.5,0.8\right]\right),\left(\left[0.6,0.9\right],\left[0.5,0.8\right].\left[0.6,0.9\right]\right)\right), \end{array}\\ \begin{array}{c} \left(e_{1},e_{3}\right),\left(u_{1},\left(\left[0.2,0.6\right],\left[0.1,0.6\right],\left[0.3,0.6\right]\right),\left(\left[0,0.2\right],\left[0.2,0.5\right],\left[0.3,0.7\right]\right),\left(\left[0.4,0.9\right],\left[0.4,0.8\right].\left[0.5,0.8\right]\right)\right),\\ \left(u_{2},\left(\left[0.2,0.5\right],\left[0.2,0.5\right],\left[0.5,0.8\right]\right),\left(\left[0.3,0.5\right],\left[0.3,0.5\right],\left[0.5,0.8\right]\right),\left(\left[0.5,0.9\right],\left[0.5,0.9\right].\left[0.6,0.9\right]\right)\right), \end{array} \end{array}\right\}. \end{array}$$

**Definition 26 .**

Let *℘*_*ℜ*_ be an mPIVNSS. Then, necessity operation on mIVPNSS is represented by ⊕*℘*_*ℜ*_ and defined as follows:

(42) $$\begin{array}{c} ⊕{℘}_{ℜ}=\left\{\begin{array}{c} \left(e,\left\{\left(\begin{array}{c} u,\;\left[u_{\alpha }^{\ell ℜ}\left(u\right),u_{\alpha }^{uℜ}\left(u\right)\right],\;\left[v_{\alpha }^{\ell ℜ}\left(u\right),v_{\alpha }^{uℜ}\left(u\right)\right],\\ \left[1-u_{\alpha }^{uℜ}\left(u\right),1-u_{\alpha }^{\ell ℜ}\left(u\right)\right] \end{array}\right):u\in U,\alpha =1,2,3,\ldots ,m\right\}\right):e\in E \end{array}\right\}. \end{array}$$

**Definition 27 .**

Let *℘*_*ℜ*_ be the mPIVNSS over *𝒰*. Then, possibility operation on mIVPNSS is represented by ⊗*℘*_*ℜ*_ and defined as follows:

(43) $$\begin{array}{c} ⊗{℘}_{ℜ}=\left\{\begin{array}{c} \left(e,\;\left\{\left(\begin{array}{c} u,\left[1-w_{\alpha }^{uℜ}\left(u\right),1-w_{\alpha }^{\ell ℜ}\left(u\right)\right],\;\\ \left[v_{\alpha }^{\ell ℜ}\left(u\right),\;v_{\alpha }^{uℜ}\left(u\right)\right],\left[w_{\alpha }^{\ell ℜ}\left(u\right),\;w_{\alpha }^{uℜ}\left(u\right)\right] \end{array}\right):u\in U,\alpha =1,2,3,\ldots ,m\right\}\right):e\in E \end{array}\right\}. \end{array}$$

**Proposition 4 .**

Let *℘*_*ℜ*_ and *℘*_*ℒ*_ be two mPIVNSSs over *𝒰*. Then,

(44) $$\begin{array}{c} ⊕\left({℘}_{ℜ}\cup_{\epsilon }{℘}_{L}\right)=⊕{℘}_{L}\cup_{\epsilon }⊕{℘}_{ℜ},\\ ⊕\left({℘}_{ℜ}\cap_{\epsilon }{℘}_{L}\right)=⊕{℘}_{L}\cap_{\epsilon }⊕{℘}_{ℜ}. \end{array}$$

**Proof**

As we know,

(45) $$\begin{array}{c} {℘}_{ℜ}=\left\{\begin{array}{c} \left(e,\left\{\left(u,\left[u_{\alpha }^{\ell ℜ}\left(u\right),u_{\alpha }^{uℜ}\left(u\right)\right],\left[v_{\alpha }^{\ell ℜ}\left(u\right),v_{\alpha }^{uℜ}\left(u\right)\right],\left[w_{\alpha }^{\ell ℜ}\left(u\right),w_{\alpha }^{uℜ}\left(u\right)\right]\right):u\in U,\alpha =1,2,3,\ldots ,m\right\}\right):e\in E \end{array}\right\},\\ {℘}_{L}\left(e\right)=\left\{\begin{array}{c} \left(e,\left\{\left(u,\left[u_{\alpha }^{\ell L}\left(u\right),u_{\alpha }^{uL}\left(u\right)\right],\left[v_{\alpha }^{\ell L}\left(u\right),v_{\alpha }^{uL}\left(u\right)\right],\left[w_{\alpha }^{\ell L}\left(u\right),w_{\alpha }^{uL}\left(u\right)\right]\right):u\in U,\alpha =1,2,3,\ldots ,m\right\}\right):e\in E \end{array}\right\}, \end{array}$$

be two mPIVNSSs over *𝒰*.

Let *℘*_*ℜ*_∪_*ϵ*_*℘*_*ℒ*_=*℘*_*ℋ*_:

(46) $$\begin{array}{c} u\left({℘}_{H}\right)=\left\{\begin{array}{cc} \left[u_{\alpha }^{\ell R}\left(u\right),\;u_{\alpha }^{uR}\left(u\right)\right], & \text{if }e\in ℜ-L,\\ \left[u_{\alpha }^{\ell L}\left(u\right),\;u_{\alpha }^{uL}\left(u\right)\right], & \text{if }e\in L-ℜ,\\ \left[\max\left\{u_{\alpha }^{\ell R}\left(u\right),u_{\alpha }^{\ell L}\left(u\right)\right\},\text{max}\left\{u_{\alpha }^{uR}\left(u\right),u_{\alpha }^{uL}\left(u\right)\right\}\right], & \text{if }e\in ℜ\cap L, \end{array}\right.\\ v\left({℘}_{H}\right)=\left\{\begin{array}{cc} \left[v_{\alpha }^{\ell R}\left(u\right),\;v_{\alpha }^{uR}\left(u\right)\right], & \text{if }e\in ℜ-L,\\ \left[v_{\alpha }^{\ell L}\left(u\right),\;v_{\alpha }^{uL}\left(u\right)\right], & \text{if }e\in L-ℜ,\\ \left[\min\left\{v_{\alpha }^{\ell R}\left(u\right),v_{\alpha }^{\ell L}\left(u\right)\right\},\text{min}\left\{v_{\alpha }^{uR}\left(u\right),v_{\alpha }^{uL}\left(u\right)\right\}\right], & \text{if }e\in ℜ\cap L, \end{array}\right.\\ w\left({℘}_{H}\right)=\left\{\begin{array}{cc} \left[w_{\alpha }^{\ell R}\left(u\right),w_{\alpha }^{uR}\left(u\right)\right], & \text{if }e\in ℜ-L,\\ \left[w_{\alpha }^{\ell L}\left(u\right),w_{\alpha }^{uL}\left(u\right)\right], & \text{if }e\in L-ℜ,\\ \left[\min\left\{w_{\alpha }^{\ell R}\left(u\right),w_{\alpha }^{\ell L}\left(u\right)\right\},\min\left\{w_{\alpha }^{uR}\left(u\right),w_{\alpha }^{uL}\left(u\right)\right\}\right], & \text{if }e\in ℜ\cap L. \end{array}\right. \end{array}$$

By using Definition 26,

(47) $$\begin{array}{c} ⊕u\left({℘}_{H}\right)=\left\{\begin{array}{cc} \left[u_{\alpha }^{\ell R}\left(u\right),\;u_{\alpha }^{uR}\left(u\right)\right], & \text{if }e\in ℜ-L,\\ \left[u_{\alpha }^{\ell L}\left(u\right),\;u_{\alpha }^{uL}\left(u\right)\right], & \text{if }e\in L-ℜ,\\ \left[\max\left\{u_{\alpha }^{\ell R}\left(u\right),u_{\alpha }^{\ell L}\left(u\right)\right\},\text{max}\left\{u_{\alpha }^{uR}\left(u\right),u_{\alpha }^{uL}\left(u\right)\right\}\right], & \text{if }e\in ℜ\cap L, \end{array}\right.\\ ⊕v\left({℘}_{H}\right)=\left\{\begin{array}{cc} \left[v_{\alpha }^{\ell R}\left(u\right),\;v_{\alpha }^{uR}\left(u\right)\right], & \text{if }e\in ℜ-L,\\ \left[v_{\alpha }^{\ell L}\left(u\right),\;v_{\alpha }^{uL}\left(u\right)\right], & \text{if }e\in L-ℜ,\\ \left[\min\left\{v_{\alpha }^{\ell R}\left(u\right),v_{\alpha }^{\ell L}\left(u\right)\right\},\text{min}\left\{v_{\alpha }^{uR}\left(u\right),v_{\alpha }^{uL}\left(u\right)\right\}\right], & \text{if }e\in ℜ\cap L, \end{array}\right.\\ ⊕w\left({℘}_{H}\right)=\left\{\begin{array}{cc} \left[1-u_{\alpha }^{\ell R}\left(u\right),1-u_{\alpha }^{uR}\left(u\right)\right], & \text{if }e\in ℜ-L,\\ \left[1-u_{\alpha }^{\ell L}\left(u\right),1-u_{\alpha }^{uL}\left(u\right)\right], & \text{if }e\in L-ℜ,\\ \left[\min\left\{u1{-}_{\alpha }^{\ell R}\left(u\right),1-u_{\alpha }^{\ell L}\left(u\right)\right\},\min\left\{1-u_{\alpha }^{uR}\left(u\right),1-u_{\alpha }^{uL}\left(u\right)\right\}\right], & \text{if }e\in ℜ\cap L. \end{array}\right. \end{array}$$

Assume ⊕*℘*_*ℒ*_∪_*ϵ*_ ⊕ *℘*_*ℜ*_=*ℵ*, where ⊕*℘*_*ℜ*_ and ⊕*℘*_*ℒ*_ are given as follows by using the definition of necessity operation.

(48) $$\begin{array}{c} ⊕{℘}_{ℜ}\left(e\right)=\left\{\begin{array}{c} \left(e,\;\left\{\left(\begin{array}{c} u,\;\left[u_{\alpha }^{\ell ℜ}\left(u\right),\;u_{\alpha }^{uℜ}\left(u\right)\right],\;\left[v_{\alpha }^{\ell ℜ}\left(u\right),v_{\alpha }^{uℜ}\left(u\right)\right],\;\\ \left[1-u_{\alpha }^{uℜ}\left(u\right),\;1-u_{\alpha }^{\ell ℜ}\left(u\right)\right] \end{array}\right):u\in U,\alpha =1,2,3,\ldots ,m\right\}\right):e\in E \end{array}\right\},\\ ⊕{℘}_{L}\left(e\right)=\left\{\begin{array}{c} \left(e,\;\left\{\left(\begin{array}{c} u,\;\left[u_{\alpha }^{\ell L}\left(u\right),\;u_{\alpha }^{uL}\left(u\right)\right],\;\left[v_{\alpha }^{\ell L}\left(u\right),\;v_{\alpha }^{uL}\left(u\right)\right],\;\\ \left[1-u_{\alpha }^{uL}\left(u\right),\;1-u_{\alpha }^{\ell L}\left(u\right)\right] \end{array}\right):u\in U,\alpha =1,2,3,\ldots ,m\right\}\right):e\in E \end{array}\right\}. \end{array}$$

By using Definition 16,

(49) $$\begin{array}{c} u\left(ℵ\right)=\left\{\begin{array}{cc} \left[u_{\alpha }^{\ell R}\left(u\right),\;u_{\alpha }^{uR}\left(u\right)\right], & \text{if }e\in ℜ-L,\\ \left[u_{\alpha }^{\ell L}\left(u\right),\;u_{\alpha }^{uL}\left(u\right)\right], & \text{if }e\in L-ℜ,\\ \left[\max\left\{1-u_{\alpha }^{\ell R}\left(u\right),1-u_{\alpha }^{\ell L}\left(u\right)\right\},\text{max}\left\{1-u_{\alpha }^{uR}\left(u\right),1-u_{\alpha }^{uL}\left(u\right)\right\}\right], & \text{if }e\in ℜ\cap L, \end{array}\right.\\ v\left(ℵ\right)=\left\{\begin{array}{cc} \left[v_{\alpha }^{\ell R}\left(u\right),\;v_{\alpha }^{uR}\left(u\right)\right], & \text{if }e\in ℜ-L,\\ \left[v_{\alpha }^{\ell L}\left(u\right),\;v_{\alpha }^{uL}\left(u\right)\right], & \text{if }e\in L-ℜ,\\ \left[\min\left\{1-v_{\alpha }^{\ell R}\left(u\right),1-v_{\alpha }^{\ell L}\left(u\right)\right\},\text{min}\left\{1-v_{\alpha }^{uR}\left(u\right),1-v_{\alpha }^{uL}\left(u\right)\right\}\right], & \text{if }e\in ℜ\cap L, \end{array}\right.\\ w\left(ℵ\right)=\left\{\begin{array}{cc} \left[1-u_{\alpha }^{\ell R}\left(u\right),1-u_{\alpha }^{uR}\left(u\right)\right], & \text{if }e\in ℜ-L,\\ \left[1-u_{\alpha }^{\ell L}\left(u\right),1-u_{\alpha }^{uL}\left(u\right)\right], & \text{if }e\in L-ℜ,\\ \left[\min\left\{1-u_{\alpha }^{\ell R}\left(u\right),1-u_{\alpha }^{\ell L}\left(u\right)\right\},\min\left\{1-u_{\alpha }^{uR}\left(u\right),1-u_{\alpha }^{uL}\left(u\right)\right\}\right], & \text{if }e\in ℜ\cap L. \end{array}\right. \end{array}$$

Therefore, (*℘*_*ℜ*_ ∪_*ϵ*_ *℘*_*ℒ*_)=⊕*℘*_*ℒ*_∪_*ϵ*_ ⊕ *℘*_*ℜ*_.

## 4. Correlation Coefficient of m-Polar Interval-Valued Neutrosophic Soft Set

In this section, we introduce CC and WCC with their properties for mPIVNSS and present the decision-making approach by using developed CC.

**Definition 28 .**

Let

(50) $$\begin{array}{c} {℘}_{ℜ}=\left\{\begin{array}{c} \left(e,\;\left\{\left(u,\left[u_{\alpha }^{\ell ℜ}\left(u_{j}\right),u_{\alpha }^{uℜ}\left(u_{j}\right)\right],\left[v_{\alpha }^{\ell ℜ}\left(u_{j}\right),v_{\alpha }^{uℜ}\left(u_{j}\right)\right],\left[w_{\alpha }^{\ell ℜ}\left(u_{j}\right),w_{\alpha }^{uℜ}\left(u_{j}\right)\right]\right):u_{j}\in U,\;\alpha =1,2,3,\ldots ,m\right\}\right):e\in E \end{array}\right\},\\ {℘}_{L}=\left\{\begin{array}{c} \left(e,\;\left\{\left(u,\left[u_{\alpha }^{\ell L}\left(u_{j}\right),u_{\alpha }^{uL}\left(u_{j}\right)\right],\left[v_{\alpha }^{\ell L}\left(u_{j}\right),v_{\alpha }^{uL}\left(u_{j}\right)\right],\left[w_{\alpha }^{\ell L}\left(u_{j}\right),w_{\alpha }^{uL}\left(u_{j}\right)\right]\right):u_{j}\in U,\;\alpha =1,2,3,\ldots ,m\right\}\right):e\in E \end{array}\right\}, \end{array}$$

be two mPIVNSSs over the universe of discourse *𝒰*. Then, informational neutrosophic energies for mPIVNSS can be presented as

(51) $$\begin{array}{c} \varsigma_{\text{mPIVNSS}}\left({℘}_{ℜ}\right)=\sum_{\alpha =1}^{m}\sum_{j=1}^{n}\left({\left(u_{\alpha }^{\ell ℜ}\left(u_{j}\right)\right)}^{2}+{\left(u_{\alpha }^{uℜ}\left(u_{j}\right)\right)}^{2}+{\left(v_{\alpha }^{\ell ℜ}\left(u_{j}\right)\right)}^{2}+{\left(v_{\alpha }^{uℜ}\left(u_{j}\right)\right)}^{2}+{\left(w_{\alpha }^{\ell ℜ}\left(u_{j}\right)\right)}^{2}+{\left(w_{\alpha }^{uℜ}\left(u_{j}\right)\right)}^{2}\right),\\ \varsigma_{\text{mPIVNSS}}\left({℘}_{L}\right)=\overset{m}{\underset{\alpha =1}{\sum }}\overset{n}{\underset{j=1}{\sum }}\left({\left(u_{\alpha }^{\ell L}\left(u_{j}\right)\right)}^{2}+{\left(u_{\alpha }^{uL}\left(u_{j}\right)\right)}^{2}+{\left(v_{\alpha }^{\ell L}\left(u_{j}\right)\right)}^{2}+{\left(v_{\alpha }^{uL}\left(u_{j}\right)\right)}^{2}+{\left(w_{\alpha }^{\ell L}\left(u_{j}\right)\right)}^{2}+{\left(w_{\alpha }^{uL}\left(u_{j}\right)\right)}^{2}\right). \end{array}$$

**Definition 29 .**

Let *℘*_*R*_ and *℘*_*ℒ*_ be two mPIVNSSs. Then, the correlation between them defined as

(52) $$\begin{array}{c} C_{\text{mPIVNSS}}\left({℘}_{ℜ},\;{℘}_{L}\right)=\sum_{\alpha =1}^{m}\sum_{j=1}^{n}\left(u_{\alpha }^{\ell ℜ}\left(u_{j}\right)*u_{\alpha }^{\ell L}\left(u_{j}\right)+\;u_{\alpha }^{uℜ}\left(u_{j}\right)*u_{\alpha }^{uL}\left(u_{j}\right)+v_{\alpha }^{\ell ℜ}\left(u_{j}\right)*v_{\alpha }^{\ell L}\left(u_{j}\right)+\;v_{\alpha }^{uℜ}\left(u_{j}\right)*v_{\alpha }^{uL}\left(u_{j}\right)+w_{\alpha }^{\ell ℜ}\left(u_{j}\right)*w_{\alpha }^{\ell L}\left(u_{j}\right)+\;w_{\alpha }^{uℜ}\left(u_{j}\right)*w_{\alpha }^{uL}\left(u_{j}\right)\right). \end{array}$$

**Theorem 1 .**

Let *℘*_*ℜ*_ and *℘*_*ℒ*_ be two mPIVNSSs and *𝒞*_mPIVNSS_(*℘*_*ℜ*_, *℘*_*ℒ*_) represents the correlation among them. Then, the subsequent estates hold.

1. *𝒞*_mPIVNSS_(*℘*_*ℜ*_, *℘*_*ℜ*_)=*ς*_mPIVNSS_(*℘*_*ℜ*_)
2. *𝒞*_mPIVNSS_(*℘*_*ℒ*_, *℘*_*ℒ*_)=*ς*_mPIVNSS_(*℘*_*ℒ*_)

**Proof**

The proof is trivial.

**Definition 30 .**

Let *℘*_*ℜ*_ and *℘*_*ℒ*_ be two mPIVNSSs over *𝒰*. Then, the CC between them is given as *δ*_mPIVNSS_(*℘*_*ℜ*_, *℘*_*ℒ*_) and can be stated as follows:

(53) $$\begin{array}{c} \delta_{\text{mPIVNSS}}\left({℘}_{R},{℘}_{L}\right)=\frac{C_{\text{mPIVNSS}}\left({℘}_{R},{℘}_{L}\right)\;}{\sqrt{\varsigma_{\text{mPIVNSS}}\left({℘}_{R}\right)*\varsigma_{\text{mPIVNSS}}\left({℘}_{L}\right)}}, \end{array}$$

(54) $$\begin{array}{c} \delta_{\text{mPIVNSS}}\left({℘}_{ℜ},{℘}_{L}\right)=\frac{\sum_{\alpha =1}^{m}\sum_{j=1}^{n}\left(\begin{array}{c} u_{\alpha }^{\ell ℜ}\left(u_{j}\right)*u_{\alpha }^{\ell L}\left(u_{j}\right)+u_{\alpha }^{uℜ}\left(u_{j}\right)*u_{\alpha }^{uL}\left(u_{j}\right)+v_{\alpha }^{\ell ℜ}\left(u_{j}\right)*v_{\alpha }^{\ell L}\left(u_{j}\right)+\\ v_{\alpha }^{uℜ}\left(u_{j}\right)*v_{\alpha }^{uL}\left(u_{j}\right)+w_{\alpha }^{\ell ℜ}\left(u_{j}\right)*w_{\alpha }^{\ell L}\left(u_{j}\right)+w_{\alpha }^{uℜ}\left(u_{j}\right)*w_{\alpha }^{uL}\left(u_{j}\right) \end{array}\right)}{\left(\begin{array}{c} \sqrt{\sum_{\alpha =1}^{m}\sum_{j=1}^{n}\left({\left(u_{\alpha }^{\ell ℜ}\left(u_{j}\right)\right)}^{2}+{\left(u_{\alpha }^{uℜ}\left(u_{j}\right)\right)}^{2}+{\left(v_{\alpha }^{\ell ℜ}\left(u_{j}\right)\right)}^{2}+{\left(v_{\alpha }^{uℜ}\left(u_{j}\right)\right)}^{2}+{\left(w_{\alpha }^{\ell ℜ}\left(u_{j}\right)\right)}^{2}+{\left(w_{\alpha }^{uℜ}\left(u_{j}\right)\right)}^{2}\right)}\\ \sqrt{\sum_{\alpha =1}^{m}\sum_{j=1}^{n}\left({\left(u_{\alpha }^{\ell L}\left(u_{j}\right)\right)}^{2}+{\left(u_{\alpha }^{uL}\left(u_{j}\right)\right)}^{2}+{\left(v_{\alpha }^{\ell L}\left(u_{j}\right)\right)}^{2}+{\left(v_{\alpha }^{uL}\left(u_{j}\right)\right)}^{2}+{\left(w_{\alpha }^{\ell L}\left(u_{j}\right)\right)}^{2}+{\left(w_{\alpha }^{uL}\left(u_{j}\right)\right)}^{2}\right)} \end{array}\right)}. \end{array}$$

**Theorem 2 .**

Let *℘*_*ℜ*_ and *℘*_*ℒ*_ be two mPIVNSSs over *𝒰*. Then, CC between them satisfies the following properties.

1. 0 ≤ *δ*_mPIVNSS_(*℘*_*ℜ*_,  *℘*_*ℒ*_) ≤ 1
2. *δ*_mPIVNSS_(*℘*_*ℜ*_, *℘*_*ℒ*_)=*δ*_mPIVNSS_(*℘*_*ℒ*_, *℘*_*ℜ*_)
3. If *℘*_*ℜ*_=*℘*_*ℒ*_, that is, ∀*j*, *α*, *u*_*α*_^*ℓℜ*^(*u*_*j*_)=*u*_*α*_^*ℓℒ*^(*u*_*j*_), *u*_*α*_^*𝔲ℜ*^(*u*_*j*_)=*u*_*α*_^*𝔲ℒ*^(*u*_*j*_), *v*_*α*_^*ℓℜ*^(*u*_*j*_)=*v*_*α*_^*ℓℒ*^(*u*_*j*_), *v*_*α*_^*𝔲ℜ*^(*u*_*j*_)=*v*_*α*_^*𝔲ℒ*^(*u*_*j*_), *w*_*α*_^*ℓℜ*^(*u*_*j*_)=*w*_*α*_^*ℓℒ*^(*u*_*j*_), *w*_*α*_^*𝔲ℜ*^(*u*_*j*_)=*w*_*α*_^*𝔲ℒ*^(*u*_*j*_), then *δ*_mPIVNSS_(*℘*_*ℜ*_, *℘*_*ℒ*_)=1.

**Proof**

The proof is obvious.

**Definition 31 .**

Let *℘*_*ℜ*_ and *℘*_*ℒ*_ be two mPIVNSSs over *𝒰*. Then, their CC has also been given as *δ*_mPIVNSS_^1^(*℘*_*ℜ*_, *℘*_*ℒ*_) and is expressed as follows:

(55) $$\begin{array}{c} \delta_{\text{mPIVNSS}}^{1}\left({℘}_{ℜ},\;{℘}_{L}\right)=\frac{C_{\text{mPIVNSS}}\left({℘}_{ℜ},\;{℘}_{L}\right)\;}{\max\left\{\varsigma_{\text{mPIVNSS}}\left({℘}_{ℜ}\right),\;\varsigma_{\text{mPIVNSS}}\left({℘}_{L}\right)\right\}},\\ \delta_{\text{IVIFSS}}^{1}\left({℘}_{ℜ},{℘}_{L}\right)=\frac{\sum_{\alpha =1}^{m}\sum_{j=1}^{n}\left(\begin{array}{c} u_{\alpha }^{\ell ℜ}\left(u_{j}\right)*u_{\alpha }^{\ell L}\left(u_{j}\right)+u_{\alpha }^{uℜ}\left(u_{j}\right)*u_{\alpha }^{uL}\left(u_{j}\right)+v_{\alpha }^{\ell ℜ}\left(u_{j}\right)*v_{\alpha }^{\ell L}\left(u_{j}\right)+\\ v_{\alpha }^{uℜ}\left(u_{j}\right)*v_{\alpha }^{uL}\left(u_{j}\right)+w_{\alpha }^{\ell ℜ}\left(u_{j}\right)*w_{\alpha }^{\ell L}\left(u_{j}\right)+w_{\alpha }^{uℜ}\left(u_{j}\right)*w_{\alpha }^{uL}\left(u_{j}\right) \end{array}\right)}{\max\left\{\begin{array}{c} \sqrt{\sum_{\alpha =1}^{m}\sum_{j=1}^{n}\left({\left(u_{\alpha }^{\ell ℜ}\left(u_{j}\right)\right)}^{2}+{\left(u_{\alpha }^{uℜ}\left(u_{j}\right)\right)}^{2}+{\left(v_{\alpha }^{\ell ℜ}\left(u_{j}\right)\right)}^{2}+{\left(v_{\alpha }^{uℜ}\left(u_{j}\right)\right)}^{2}+{\left(w_{\alpha }^{\ell ℜ}\left(u_{j}\right)\right)}^{2}+{\left(w_{\alpha }^{uℜ}\left(u_{j}\right)\right)}^{2}\right)}\\ \sqrt{\sum_{\alpha =1}^{m}\sum_{j=1}^{n}\left({\left(u_{\alpha }^{\ell L}\left(u_{j}\right)\right)}^{2}+{\left(u_{\alpha }^{uL}\left(u_{j}\right)\right)}^{2}+{\left(v_{\alpha }^{\ell L}\left(u_{j}\right)\right)}^{2}+{\left(v_{\alpha }^{uL}\left(u_{j}\right)\right)}^{2}+{\left(w_{\alpha }^{\ell L}\left(u_{j}\right)\right)}^{2}+{\left(w_{\alpha }^{uL}\left(u_{j}\right)\right)}^{2}\right)} \end{array}\right\}}. \end{array}$$

**Theorem 3 .**

Let *℘*_*ℜ*_ and *℘*_*ℒ*_ be two mPIVNSSs over *𝒰*. Then, CC between them satisfies the following properties.

1. 0 0 ≤ *δ*_IVIFSS_^1^(*℘*_*ℜ*_, *℘*_*ℒ*_) ≤ 1
2. *δ*_IVIFSS_^1^(*℘*_*ℜ*_, *℘*_*ℒ*_)=*δ*_IVIFSS_^1^(*℘*_*ℒ*_,  *℘*_*ℜ*_)
3. If *℘*_*ℜ*_=*℘*_*ℒ*_, that is, ∀*j*, *α*, *u*_*α*_^*ℓℜ*^(*u*_*j*_)=*u*_*α*_^*ℓℒ*^(*u*_*j*_), *u*_*α*_^*𝔲ℜ*^(*u*_*j*_)=*u*_*α*_^*𝔲ℒ*^(*u*_*j*_), *v*_*α*_^*ℓℜ*^(*u*_*j*_)=*v*_*α*_^*ℓℒ*^(*u*_*j*_), *v*_*α*_^*𝔲ℜ*^(*u*_*j*_)=*v*_*α*_^*𝔲ℒ*^(*u*_*j*_), *w*_*α*_^*ℓℜ*^(*u*_*j*_)=*w*_*α*_^*ℓℒ*^(*u*_*j*_), *w*_*α*_^*𝔲ℜ*^(*u*_*j*_)=*w*_*α*_^*𝔲ℒ*^(*u*_*j*_), then *δ*_IVIFSS_^1^(*℘*_*ℜ*_,  *℘*_*ℒ*_)=1.

**Proof**

The proof is obvious.

These days, it is important to discuss the weight of mPNSS for practical life. When professionals set different weights for each alternative, the decision may be different. Therefore, it is perfectly correct for experts to weigh the recent decision. Suppose the weight of professionals can be stated as $\overset{ˇ}{\omega }={\left\{{\overset{ˇ}{\omega }}_{1},{\overset{ˇ}{\omega }}_{2},{\overset{ˇ}{\omega }}_{3},\ldots ,{\overset{ˇ}{\omega }}_{m}\right\}}^{T}$, where ${\overset{ˇ}{\omega }}_{k}>0$, $\sum_{k=1}^{m}{\overset{ˇ}{\omega }}_{k}=1$. The weights for an attribute can be assumed as follows: *γ*={*γ*_1_, *γ*_2_, *γ*_3_,…,*γ*_*n*_}^*T*^, where *γ*_*i*_ > 0, ∑_*i*=1_^*n*^*γ*_*i*_=1.

**Definition 32 .**

Let *℘*_*ℜ*_ and *℘*_*ℒ*_ are two mPIVNSS over *𝒰*. Then, their WCC is given as *δ*_WmPIVNSS_(*℘*_*ℜ*_, *℘*_*ℒ*_) and stated as follows:

(56) $$\begin{array}{c} \delta_{\text{WmPIVNSS}}\left({℘}_{R},{℘}_{L}\right)=\frac{C_{\text{mPIVNSS}}\left({℘}_{R},{℘}_{L}\right)\;}{\sqrt{\varsigma_{\text{mPIVNSS}}\left({℘}_{R}\right)*\varsigma_{\text{mPIVNSS}}\left({℘}_{L}\right)}},\\ \delta_{\text{WmPIVNSS}}\left({℘}_{R},{℘}_{L}\right)=\frac{\sum_{\alpha ,k=1}^{m}\overset{ˇ}{\omega }\left(\sum_{i,j=1}^{n}\gamma_{i}\left(\begin{array}{c} u_{\alpha }^{\ell ℜ}\left(u_{j}\right)*u_{\alpha }^{\ell L}\left(u_{j}\right)+u_{\alpha }^{uℜ}\left(u_{j}\right)*u_{\alpha }^{uL}\left(u_{j}\right)+v_{\alpha }^{\ell ℜ}\left(u_{j}\right)*v_{\alpha }^{\ell L}\left(u_{j}\right)+\\ v_{\alpha }^{uℜ}\left(u_{j}\right)*v_{\alpha }^{uL}\left(u_{j}\right)+w_{\alpha }^{\ell ℜ}\left(u_{j}\right)*w_{\alpha }^{\ell L}\left(u_{j}\right)+w_{\alpha }^{uℜ}\left(u_{j}\right)*w_{\alpha }^{uL}\left(u_{j}\right) \end{array}\right)\right)}{\left(\begin{array}{c} \sqrt{\sum_{\alpha ,k=1}^{m}\overset{ˇ}{\omega }\left(\sum_{i,j=1}^{n}\gamma_{i}\left({\left(u_{\alpha }^{\ell ℜ}\left(u_{j}\right)\right)}^{2}+{\left(u_{\alpha }^{uℜ}\left(u_{j}\right)\right)}^{2}+{\left(v_{\alpha }^{\ell ℜ}\left(u_{j}\right)\right)}^{2}+{\left(v_{\alpha }^{uℜ}\left(u_{j}\right)\right)}^{2}+{\left(w_{\alpha }^{\ell ℜ}\left(u_{j}\right)\right)}^{2}+{\left(w_{\alpha }^{uℜ}\left(u_{j}\right)\right)}^{2}\right)\right)}\\ \sqrt{\sum_{\alpha ,k=1}^{m}\overset{ˇ}{\omega }\left(\sum_{i,j=1}^{n}\gamma_{i}\left({\left(u_{\alpha }^{\ell L}\left(u_{j}\right)\right)}^{2}+{\left(u_{\alpha }^{uL}\left(u_{j}\right)\right)}^{2}+{\left(v_{\alpha }^{\ell L}\left(u_{j}\right)\right)}^{2}+{\left(v_{\alpha }^{uL}\left(u_{j}\right)\right)}^{2}+{\left(w_{\alpha }^{\ell L}\left(u_{j}\right)\right)}^{2}+{\left(w_{\alpha }^{uL}\left(u_{j}\right)\right)}^{2}\right)\right)} \end{array}\right)}. \end{array}$$

**Definition 33 .**

Let *℘*_*ℜ*_ and *℘*_*ℒ*_ be two mPIVNSSs over *𝒰*. Then, their WCC also given as *δ*_WmPIVNSS_^1^(*℘*_*ℜ*_, *℘*_*ℒ*_) is defined as follows:

(57) $$\begin{array}{c} \delta_{\text{WmPIVNSS}}^{1}\left({℘}_{R},{℘}_{L}\right)=\frac{C_{\text{mPIVNSS}}\left({℘}_{R},{℘}_{L}\right)\;}{\text{max}\left\{\varsigma_{\text{mPIVNSS}}\left({℘}_{R}\right),\varsigma_{\text{mPIVNSS}}\left({℘}_{L}\right)\right\}},\\ \delta_{\text{WmPIVNSS}}^{1}\left({℘}_{R},{℘}_{L}\right)=\frac{\sum_{\alpha ,k=1}^{m}\overset{ˇ}{\omega }\left(\sum_{i,j=1}^{n}\gamma_{i}\left(\begin{array}{c} u_{\alpha }^{\ell ℜ}\left(u_{j}\right)*u_{\alpha }^{\ell L}\left(u_{j}\right)+u_{\alpha }^{uℜ}\left(u_{j}\right)*u_{\alpha }^{uL}\left(u_{j}\right)+v_{\alpha }^{\ell ℜ}\left(u_{j}\right)*v_{\alpha }^{\ell L}\left(u_{j}\right)+\\ v_{\alpha }^{uℜ}\left(u_{j}\right)*v_{\alpha }^{uL}\left(u_{j}\right)+w_{\alpha }^{\ell ℜ}\left(u_{j}\right)*w_{\alpha }^{\ell L}\left(u_{j}\right)+w_{\alpha }^{uℜ}\left(u_{j}\right)*w_{\alpha }^{uL}\left(u_{j}\right) \end{array}\right)\right)}{\max\left\{\begin{array}{c} \sqrt{\sum_{\alpha ,k=1}^{m}\overset{ˇ}{\omega }\left(\sum_{i,j=1}^{n}\gamma_{i}\left({\left(u_{\alpha }^{\ell ℜ}\left(u_{j}\right)\right)}^{2}+{\left(u_{\alpha }^{uℜ}\left(u_{j}\right)\right)}^{2}+{\left(v_{\alpha }^{\ell ℜ}\left(u_{j}\right)\right)}^{2}+{\left(v_{\alpha }^{uℜ}\left(u_{j}\right)\right)}^{2}+{\left(w_{\alpha }^{\ell ℜ}\left(u_{j}\right)\right)}^{2}+{\left(w_{\alpha }^{uℜ}\left(u_{j}\right)\right)}^{2}\right)\right)}\\ \sqrt{\sum_{\alpha ,k=1}^{m}\overset{ˇ}{\omega }\left(\sum_{i,j=1}^{n}\gamma_{i}\left({\left(u_{\alpha }^{\ell L}\left(u_{j}\right)\right)}^{2}+{\left(u_{\alpha }^{uL}\left(u_{j}\right)\right)}^{2}+{\left(v_{\alpha }^{\ell L}\left(u_{j}\right)\right)}^{2}+{\left(v_{\alpha }^{uL}\left(u_{j}\right)\right)}^{2}+{\left(w_{\alpha }^{\ell L}\left(u_{j}\right)\right)}^{2}+{\left(w_{\alpha }^{uL}\left(u_{j}\right)\right)}^{2}\right)\right)} \end{array}\right\}}. \end{array}$$

If we consider $\overset{ˇ}{\omega }=\left\{\left(1/m\right),\left(1/m\right),\ldots ,\left(1/m\right)\right\}$ and *γ*={(1/*n*), (1/*n*),…, (1/*n*)}, then *δ*_WmPIVNSS_(*℘*_*ℜ*_, *℘*_*ℒ*_) and *δ*_WmPIVNSS_^1^(*℘*_*ℜ*_, *℘*_*ℒ*_) are reduced to *δ*_mPIVNSS_(*℘*_*ℜ*_, *℘*_*ℒ*_) and *δ*_mPIVNSS_^1^(*℘*_*ℜ*_, *℘*_*ℒ*_), respectively, defined in Definitions 30 and 31.

**Theorem 4 .**

Let *℘*_*ℜ*_ and *℘*_*ℒ*_ be two mPIVNSSs over *𝒰*. Then, WCC between them satisfies the following properties.

1. 0 ≤ *δ*_WmPIVNSS_(*℘*_*ℜ*_, *℘*_*ℒ*_) ≤ 1
2. *δ*_WmPIVNSS_(*℘*_*ℜ*_,  *℘*_*ℒ*_)=*δ*_WmPIVNSS_( *℘*_*ℒ*_,  *℘*_*ℜ*_)
3. If *℘*_*ℜ*_=*℘*_*ℒ*_, that is, ∀*j*, *α*, *u*_*α*_^*ℓℜ*^(*u*_*j*_)=*u*_*α*_^*ℓℒ*^(*u*_*j*_), *u*_*α*_^*𝔲ℜ*^(*u*_*j*_)=*u*_*α*_^*𝔲ℒ*^(*u*_*j*_), *v*_*α*_^*ℓℜ*^(*u*_*j*_)=*v*_*α*_^*ℓℒ*^(*u*_*j*_), *v*_*α*_^*𝔲ℜ*^(*u*_*j*_)=*v*_*α*_^*𝔲ℒ*^(*u*_*j*_), *w*_*α*_^*ℓℜ*^(*u*_*j*_)=*w*_*α*_^*ℓℒ*^(*u*_*j*_), *w*_*α*_^*𝔲ℜ*^(*u*_*j*_)=*w*_*α*_^*𝔲ℒ*^(*u*_*j*_), then *δ*_WmPIVNSS_(*℘*_*ℜ*_, *℘*_*ℒ*_)=1.

**Proof**

The proof is obvious.

### 4.1. Decision-Making Approach Based on Correlation Coefficient of mPIVNSS

Assume a set of “s” alternatives such as *β*={*β*^1^, *β*^2^, *β*^3^,…, *β*^*s*^} for assessment under the team of experts such as *𝒰*={*u*_1_, *u*_2_, *u*_3_,…, *u*_*n*_} with weights Ω=(Ω_1_,  Ω_1_,…, Ω_*n*_)^*T*^, such that Ω_*i*_ > 0, ∑_*i*=1_^*n*^Ω_*i*_=1. Let *ℰ*={*e*_1_, *e*_2_,…, *e*_*m*_} be a set of attributes with weights, and *γ*=(*γ*_1_, *γ*_2_, *γ*_3_,…,*γ*_*m*_)^*T*^ be a weight vector for parameters such as *γ*_*i*_ > 0, ∑_*j*=1_^*m*^*γ*_*j*_=1. The team of experts {*u*_*i*_: *i*=1,2,…, *n*} evaluate the alternatives {*β*^(*z*)^: *z*=1,2,…, *s*} under the considered parameters {*e*_*j*_: *j*=1,2,…, *m*} given in the form of mPIVNSNs *ℒ*_*ij*_^(*z*)^=(*u*_*α*_*ij*__^(*z*)^,  *v*_*α*_*ij*__^(*z*)^,  *w*_*α*_*ij*__^(*z*)^), where *u*_*α*_*ij*__^(z)^=[*u*_*α*_*ij*__^*ℓ*^(*u*), *u*_*α*_*ij*__^*𝔲*^(*u*)], *v*_*α*_*ij*__^(*z*)^=[*v*_*α*_*ij*__^*ℓ*^(*u*),  *v*_*α*_*ij*__^*𝔲*^(*u*)], and *w*_*α*_*ij*__^(*z*)^=[*w*_*α*_*ij*__^*ℓ*^(*u*), *w*_*α*_*ij*__^*𝔲*^(*u*)], where 0 ≤ *u*_*α*_^*ℓ*^(*u*), *u*_*α*_^*𝔲*^(*u*), *v*_*α*_^*ℓ*^(*u*), *v*_*α*_^*𝔲*^(*u*), *w*_*α*_^*ℓ*^(*u*), *w*_*α*_^*𝔲*^(*u*) ≤ 1 and 0 ≤ *u*_*α*_*ij*__^*𝔲*^(*u*)+*v*_*α*_*ij*__^*𝔲*^(*u*)+*w*_*α*_*ij*__^*𝔲*^(*u*) ≤ 3. So, *ℒ*_ij_^(z)^=([*u*_*α*_*ij*__^*ℓ*^(*u*), *u*_*α*_*ij*__^*𝔲*^(*u*)), [*v*_*α*_*ij*__^*ℓ*^(*u*), *v*_*α*_*ij*__^*𝔲*^(*u*)], [*w*_*α*_*ij*__^*ℓ*^(*u*), *w*_*α*_*ij*__^*𝔲*^(*u*)]) for all *i*, *j*.

**Figure 1:**
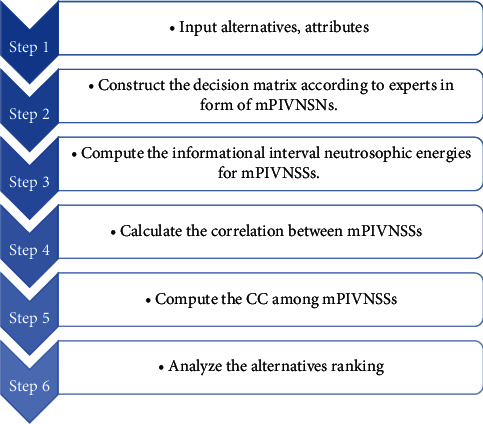
Flowchart for correlation coefficient under mPIVNSS.

The flowchart of the offered algorithm is shown in Figure 1.

## 5. Similarity Measures and Weighted Average Operator for m-Polar Interval-Valued Neutrosophic Soft Set

In the past few years, many mathematicians developed various methodologies to solve MCDM problems, such as aggregation operators for different hybrid structures, CC, similarity measures, and decision-making applications. Some operational laws and mPIVNSWA with its decision-making approach have been established for mPIVNSS which is an extension of the interval-valued neutrosophic weighted aggregation operator [47]. The idea of the score, accuracy, and certainty functions based on [48] introduces in the following section for comparing m-polar interval-valued neutrosophic numbers (mPIVNNs). We also present two different types of similarity measures with their decision-making approaches, such as cosine and set-theoretic based on Bhattacharya's distance [49, 50] for mPIVNSS.

**Definition 34 .**

Let *℘*_*ℜ*_=[*u*_*α*_^*ℓ*^(*u*), *u*_*α*_^*𝔲*^(*u*)], [*v*_*α*_^*ℓ*^(*u*),  *v*_*α*_^*𝔲*^(*u*)],  [*w*_*α*_^*ℓ*^(*u*),  *w*_*α*_^*𝔲*^(*u*)], *℘*_*ℜ*_1__=[*u*_*α*_^*ℓℜ*_1_^(*u*), *u*_*α*_^*𝔲ℜ*_1_^(*u*)], [*v*_*α*_^*ℓℜ*_1_^(*u*),  *v*_*α*_^*𝔲ℜ*_1_^(*u*)],  [*w*_*α*_^*ℓℜ*_1_^(*u*),  *w*_*α*_^*𝔲ℜ*_1_^(*u*)], and *℘*_*ℜ*_2__=[*u*_*α*_^*ℓℜ*_2_^(*u*), *u*_*α*_^*𝔲ℜ*_2_^(*u*)], [*v*_*α*_^*ℓℜ*_2_^(*u*), *v*_*α*_^*𝔲ℜ*_2_^(*u*)],  [*w*_*α*_^*ℓℜ*_2_^(*u*), *w*_*α*_^*𝔲ℜ*_2_^(*u*)] be three mPIVNSNs, and the basic operators for mPIVNSNs are defined as when *δ* > 0.

1. *℘*_*ℜ*_1__ ⊕ *℘*_*ℜ*_2__=〈[*u*_*α*_^*ℓℜ*_1_^(*u*)+*u*_*α*_^*ℓℜ*_2_^(*u*) − *u*_*α*_^*ℓℜ*_1_^(*u*)*u*_*α*_^*ℓℜ*_2_^(*u*),  *u*_*α*_^*𝔲ℜ*_1_^(*u*)+*u*_*α*_^*𝔲ℜ*_2_^(*u*) − *u*_*α*_^*𝔲ℜ*_1_^(*u*)*u*_*α*_^*𝔲ℜ*_2_^(*u*)], [*v*_*α*_^*ℓℜ*_1_^(*u*)*v*_*α*_^*ℓℜ*_2_^(*u*), *v*_*α*_^*𝔲ℜ*_1_^(*u*) *v*_*α*_^*𝔲ℜ*_2_^(*u*)],  [*w*_*α*_^*ℓℜ*_1_^(*u*)*w*_*α*_^*ℓℜ*_2_^(*u*), *w*_*α*_^*𝔲ℜ*_1_^(*u*) *w*_*α*_^*𝔲ℜ*_2_^(*u*)]〉
2. *℘*_*ℜ*_1__ ⊗ *℘*_*ℜ*_2__=〈[*u*_*α*_^*ℓℜ*_1_^(*u*)*u*_*α*_^*ℓℜ*_2_^(*u*),  *u*_*α*_^*𝔲ℜ*_1_^(*u*)*u*_*α*_^*𝔲ℜ*_2_^(*u*)], [*v*_*α*_^*ℓℜ*_1_^(*u*)+*v*_*α*_^*ℓℜ*_2_^(*u*) − *v*_*α*_^*ℓℜ*_1_^(*u*)*v*_*α*_^*ℓℜ*_2_^(*u*), *v*_*α*_^*𝔲ℜ*_1_^(*u*)+ *v*_*α*_^*𝔲ℜ*_2_^(*u*) − *v*_*α*_^*𝔲ℜ*_1_^(*u*)*v*_*α*_^*𝔲ℜ*_2_^(*u*)], [*w*_*α*_^*ℓℜ*_1_^(*u*)+*w*_*α*_^*ℓℜ*_2_^(*u*) − *w*_*α*_^*ℓℜ*_1_^(*u*)*w*_*α*_^*ℓℜ*_2_^(*u*), *w*_*α*_^*𝔲ℜ*_1_^(*u*)+ *w*_*α*_^*𝔲ℜ*_2_^(*u*) − *w*_*α*_^*𝔲ℜ*_1_^(*u*)*w*_*α*_^*𝔲ℜ*_2_^(*u*)]〉
3. *δ℘*_*ℜ*_=〈[1 − (1 − *u*_*α*_^*ℓℜ*^(*u*))^*δ*^, 1 − (1 − *u*_*α*_^*𝔲ℜ*^(*u*))^*δ*^], [(*v*_*α*_^*ℓℜ*^(*u*))^*δ*^, (*v*_*α*_^*𝔲ℜ*^(*u*))^*δ*^],  [(*w*_*α*_^*ℓℜ*^(*u*))^*δ*^, (*w*_*α*_^*𝔲ℜ*^(*u*))^*δ*^]〉
4. (*℘*_*ℜ*_)^*δ*^=〈[(*u*_*α*_^*ℓ*^(*u*))^*δ*^, (*u*_*α*_^*𝔲*^(*u*))^*δ*^], [1 − (1 − *v*_*α*_^*ℓℜ*^(*u*))^*δ*^, 1 − (1 − *v*_*α*_^*𝔲ℜ*^(*u*))^*δ*^], [1 − (1 − *w*_*α*_^*ℓℜ*^(*u*))^*δ*^, 1 − (1 − *w*_*α*_^*𝔲ℜ*^(*u*))^*δ*^] 〉

**Theorem 5 .**

Let *℘*_*ℜ*_, *℘*_*ℜ*_1__, and *℘*_*ℜ*_2__ be three mPIVNSNs and *δ*, *δ*_1_, *δ*_2_ > 0; then, the following laws hold.

1. *℘*_*ℜ*_1__ ⊕ *℘*_*ℜ*_2__=*℘*_*ℜ*_2__ ⊕ *℘*_*ℜ*_1__
2. *℘*_*ℜ*_1__ ⊗ *℘*_*ℜ*_2__=*℘*_*ℜ*_2__ ⊗ *℘*_*ℜ*_1__
3. *δ*(*℘*_*ℜ*_1__ ⊕ *℘*_*ℜ*_2__)=*δ℘*_*ℜ*_2__ ⊕ *δ℘*_*ℜ*_1__
4. (*℘*_*ℜ*_1__ ⊗ *℘*_*ℜ*_2__)^*δ*^=(*℘*_*ℜ*_1__)^*δ*^ ⊗ (*℘*_*ℜ*_2__)^*δ*^
5. *δ*_1_*℘*_*ℜ*_1__ ⊕ *δ*_2_*℘*_*ℜ*_1__=(*δ*_1_ ⊕ *δ*_2_)*℘*_*ℜ*_1__
6. (*℘*_*ℜ*_1__)^*δ*_1_^ ⊗ (*℘*_*ℜ*_1__)^*δ*_2_^=(*℘*_*ℜ*_1__)^*δ*_1_+*δ*_2_^
7. (*℘*_*ℜ*_ ⊕ *℘*_*ℜ*_1__) ⊕ *℘*_*ℜ*_2__=*℘*_*ℜ*_ ⊕ (*℘*_*ℜ*_1__ ⊕ *℘*_*ℜ*_2__)
8. (*℘*_*ℜ*_ ⊗ *℘*_*ℜ*_1__) ⊗ *℘*_*ℜ*_2__=*℘*_*ℜ*_ ⊗ (*℘*_*ℜ*_1__ ⊗ *℘*_*ℜ*_2__)

**Proof**

The proof of the above laws is straightforward by using Definition 28.

**Definition 35 .**

Let *℘*_*ℜe*_*ij*__=[*u*_*α*_*ij*__^*ℓℜ*^(*u*), *u*_*α*_ij__^*𝔲ℜ*^(*u*)], [*v*_*α*_*ij*__^*ℓℜ*^(*u*), *v*_*α*_*ij*__^*𝔲ℜ*^(*u*)], [*w*_*α*_*ij*__^*ℓℜ*^(*u*), *w*_*α*_*ij*__^*𝔲ℜ*^(*u*)] be a collection of mPIVNSNs. Ω_*i*_ and *γ*_*j*_ are the weight vectors for expert's and parameters, respectively, with given conditions Ω_*i*_ > 0, ∑_*i*=1_^*n*^Ω_*i*_=1, *γ*_*j*_ > 0, ∑_*j*=1_^*m*^*γ*_*j*_  = 1, where (*i* = 1,2,…, *n* and *j* = 1,2,…, *m*). Then, the mPIVNSWA operator defined as mPIVNSWA: Δ^*n*^⟶Δ is defined as follows:

(58) $$\begin{array}{c} \text{mPIVNSWA }\left({℘}_{ℜe_{11}},{℘}_{ℜe_{12}},\ldots ,\;{℘}_{ℜe_{nk}}\right)={⊕}_{j=1}^{k}\gamma_{j}\left({⊕}_{i=1}^{n}\Omega_{i}{℘}_{ℜe_{ij}}\;\right) \end{array}$$

**Theorem 6 .**

Let *℘*_*ℜe*_*ij*__=[*u*_*α*_*ij*__^*ℓℜ*^(*u*), *u*_*α*_ij__^*𝔲ℜ*^(*u*)], [*v*_*α*_*ij*__^*ℓℜ*^(*u*), *v*_*α*_*ij*__^*𝔲ℜ*^(*u*)], [*w*_*α*_*ij*__^*ℓℜ*^(*u*), *w*_*α*_*ij*__^*𝔲ℜ*^(*u*)] be a collection of mPIVNSNs, where (*i* = 1,2,…, *n* and *j* = 1,2,…, *k*), and the aggregated value is also an interval-valued neutrosophic soft number, such as

(59) $$\begin{array}{c} \text{mPIVNSWA }\left({℘}_{R{\text{e}}_{11}},\;{℘}_{R{\text{e}}_{12}},\ldots ,\;{℘}_{R{\text{e}}_{nk}}\right)=\langle \begin{array}{c} \left[1-{\prod_{\text{j}=1}^{m}\left(\prod_{\text{i}=1}^{n}{\left(1-u_{\alpha_{ij}}^{\ell R}\left(u\right)\right)}^{\Omega_{i}}\right)}^{\gamma_{j}},\;1-{\prod_{\text{j}=1}^{m}\left(\prod_{\text{i}=1}^{n}{\left(1-u_{\alpha_{ij}}^{uR}\left(u\right)\right)}^{\Omega_{i}}\right)}^{\gamma_{j}}\right],\\ \left[\;1-\left(1-{\prod_{\text{j}=1}^{m}\left(\prod_{\text{i}=1}^{n}{\left(1-v_{\alpha_{ij}}^{\ell R}\left(u\right)\right)}^{\Omega_{i}}\right)}^{\gamma_{j}}\right),\;1-\left(1-{\prod_{\text{j}=1}^{m}\left(\prod_{\text{i}=1}^{n}{\left(1-v_{\alpha_{ij}}^{uR}\left(u\right)\right)}^{\Omega_{i}}\right)}^{\gamma_{j}}\right)\right],\;\\ \left[1-\left(1-{\prod_{\text{j}=1}^{m}\left(\prod_{\text{i}=1}^{n}{\left(1-w_{\alpha_{ij}}^{\ell R}\left(u\right)\right)}^{\Omega_{i}}\right)}^{\gamma_{j}}\right),\;1-\left(1-{\prod_{\text{j}=1}^{m}\left(\prod_{\text{i}=1}^{n}{\left(1-w_{\alpha_{ij}}^{uR}\left(u\right)\right)}^{\Omega_{i}}\right)}^{\gamma_{j}}\right)\right] \end{array}\rangle . \end{array}$$

**Proof**

We can prove this easily by using IFSWA [51].

**Definition 36 .**

Let *℘*_*ℜ*_=[*u*_*α*_^*ℓ*^(*u*), *u*_*α*_^*𝔲*^(*u*)], [*v*_*α*_^*ℓ*^(*u*), *v*_*α*_^*𝔲*^(*u*)],  [*w*_*α*_^*ℓ*^(*u*), *w*_*α*_^*𝔲*^(*u*)] be an mPIVNSN; then the score, accuracy, and certainty functions for an mPIVNSN, respectively, defined as follows:

*𝕊*(*℘*_*ℜ*_)=(1/6*m*)(*u*_*α*_^*ℓ*^(*u*)+*u*_*α*_^*𝔲*^(*u*)+1 − *v*_*α*_^*ℓ*^(*u*)+1 − *v*_*α*_^*𝔲*^(*u*)+1 − *w*_*α*_^*ℓ*^(*u*)+1 − *w*_*α*_^*𝔲*^(*u*))(*℘*_*ℜ*_)
(2)*𝔸*(*℘*_*ℜ*_)=(1/4*m*)(4+*u*_*α*_^*ℓ*^(*u*)+*u*_*α*_^*𝔲*^(*u*) − *w*_*α*_^*ℓ*^(*u*) − *w*_*α*_^*𝔲*^(*u*))(*℘*_*ℜ*_)
(3)*ℂ*(*℘*_*ℜ*_)=(1/2*m*)(2+*u*_*α*_^*ℓ*^(*u*)+*u*_*α*_^*𝔲*^(*u*))(*℘*_*ℜ*_), where *α*=1,2,…, *m*

**Definition 37 .**

Let *℘*_*ℜ*_ and *℘*_*ℜ*_1__ be two mPIVNSSs. Then, the comparison approach is presented as follows:

(1)If (*℘*_*ℜ*_) > (*℘*_*ℜ*_1__), then *℘*_*ℜ*_ is superior to *℘*_*ℜ*_1__
(2)If (*℘*_*ℜ*_)=(*℘*_*ℜ*_1__) and (*℘*_*ℜ*_) > *𝔸*(*℘*_*ℜ*_1__), then *℘*_*ℜ*_ is superior to *℘*_*ℜ*_1__
(3)If *𝕊*(*℘*_*ℜ*_)=*𝕊*(*℘*_*ℜ*_1__), *𝔸*(*℘*_*ℜ*_)=*𝔸*(*℘*_*ℜ*_1__), and *ℂ*(*℘*_*ℜ*_) > *ℂ*(*℘*_*ℜ*_1__), then *℘*_*ℜ*_ is superior to *℘*_*ℜ*_1__
If *𝕊*(*℘*_*ℜ*_)=*𝕊*(*℘*_*ℜ*_1__), *𝔸*(*℘*_*ℜ*_) > *𝔸*(*℘*_*ℜ*_1__), and *ℂ*(*℘*_*ℜ*_)=*ℂ*(*℘*_*ℜ*_1__), then *℘*_*ℜ*_ is indifferent to *℘*_*ℜ*_1__ and can be denoted as *℘*_*ℜ*_ ~ *℘*_*ℜ*_1__

### 5.1. Decision-Making Approach-Based mPIVNSWA for mPIVNSS

Assume a set of “s” alternatives such as *β*={*β*^1^, *β*^2^, *β*^3^,…, *β*^*s*^} for assessment under the team of experts such as *𝒰*={*u*_1_, *u*_2_, *u*_3_,…, *u*_*n*_} with weights Ω=(Ω_1_,  Ω_1_,…, Ω_*n*_)^*T*^, such that Ω_*i*_ > 0, ∑_*i*=1_^*n*^Ω_*i*_=1. Let *ℰ*={*e*_1_, *e*_2_,…, *e*_*m*_} be a set of attributes with weights *γ*=(*γ*_1_, *γ*_2_, *γ*_3_,…,*γ*_*m*_)^*T*^ be a weight vector for parameters such as *γ*_*i*_ > 0, ∑_*j*=1_^*m*^*γ*_*j*_=1. The team of experts {*u*_*i*_: *i*=1,2,…, *n*} evaluate the alternatives {*β*^(*z*)^: *z*=1,2,…, *s*} under the considered parameters {*e*_*j*_: *j*=1,2,…, *m*} given in the form of mPIVNSNs *ℒ*_*ij*_^(*z*)^=(*u*_*α*_*ij*__^(*z*)^,  *v*_*α*_*ij*__^(*z*)^,  *w*_*α*_*ij*__^(*z*)^), where *u*_*α*_*ij*__^(z)^=[*u*_*α*_*ij*__^*ℓ*^(*u*), *u*_*α*_*ij*__^*𝔲*^(*u*)], *v*_*α*_*ij*__^(*z*)^=[*v*_*α*_*ij*__^*ℓ*^(*u*),  *v*_*α*_*ij*__^*𝔲*^(*u*)], and *w*_*α*_*ij*__^(*z*)^=[*w*_*α*_*ij*__^*ℓ*^(*u*), *w*_*α*_*ij*__^*𝔲*^(*u*)], where 0 ≤ *u*_*α*_^*ℓ*^(*u*), *u*_*α*_^*𝔲*^(*u*), *v*_*α*_^*ℓ*^(*u*), *v*_*α*_^*𝔲*^(*u*), *w*_*α*_^*ℓ*^(*u*), *w*_*α*_^*𝔲*^(*u*) ≤ 1 and 0 ≤ *u*_*α*_*ij*__^*𝔲*^(*u*)+*v*_*α*_*ij*__^*𝔲*^(*u*)+*w*_*α*_*ij*__^*𝔲*^(*u*) ≤ 3. So, Δ_*k*_=([*u*_*α*_*ij*__^*ℓ*^(*u*), *u*_*α*_*ij*__^*𝔲*^(*u*)], [*v*_*α*_*ij*__^*ℓ*^(*u*), *v*_*α*_*ij*__^*𝔲*^(*u*)], [*w*_*α*_*ij*__^*ℓ*^(*u*), *w*_*α*_*ij*__^*𝔲*^(*u*)]) for all *i*, *j*. Experts give their preferences for each alternative in term of mPIVNSNs by using the mPIVNSWA operator in the form ofΔ_*k*_=([*u*_*α*_*ij*__^*ℓ*^(*u*), *u*_*α*_*ij*__^*𝔲*^(*u*)], [*v*_*α*_*ij*__^*ℓ*^(*u*), *v*_*α*_*ij*__^*𝔲*^(*u*)], [*w*_*α*_*ij*__^*ℓ*^(*u*), *w*_*α*_*ij*__^*𝔲*^(*u*)]). Compute the score values for each alternative and analyze the ranking of the alternatives.

**Figure 2:**
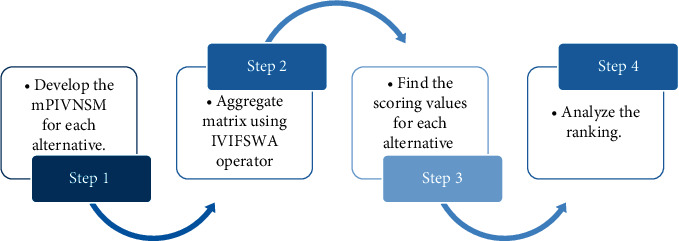
Decision-making model for mPIVNSS.

The flowchart of the offered algorithm is shown in Figure 2.

**Definition 38 .**

Let *℘*_*R*_1__ and *℘*_*R*_2__ be two mPIVNSSs over the universe of discourse *𝒰*={*u*_1_,  *u*_2_,…,  *u*_*j*_}. Then, a cosine similarity measure between *℘*_*R*_1__ and *℘*_*R*_2__ is defined as

(60) $$\begin{array}{c} S_{\text{mPIVNSS}}^{1}\left({℘}_{R_{1}},{℘}_{R_{2}}\right)=\frac{1}{mn}\overset{n}{\underset{j=1}{\sum }}\overset{m}{\underset{\alpha =1}{\sum }}\frac{\left(\begin{array}{c} \left(u_{\alpha }^{\ell ℜ}\left(u_{j}\right)+u_{\alpha }^{uℜ}\left(u_{j}\right)\right)\left(u_{\alpha }^{\ell L}\left(u_{j}\right)+\;u_{\alpha }^{uL}\left(u_{j}\right)\right)+\left(v_{\alpha }^{\ell ℜ}\left(u_{j}\right)+v_{\alpha }^{uℜ}\left(u_{j}\right)\right)\left(v_{\alpha }^{\ell L}\left(u_{j}\right)+\;v_{\alpha }^{uL}\left(u_{j}\right)\right)+\\ \left(w_{\alpha }^{\ell ℜ}\left(u_{j}\right)+w_{\alpha }^{uℜ}\left(u_{j}\right)\right)\left(w_{\alpha }^{\ell L}\left(u_{j}\right)+\;w_{\alpha }^{uL}\left(u_{j}\right)\right) \end{array}\right)}{\begin{array}{c} \left(\begin{array}{c} \sqrt{\left({\left(u_{\alpha }^{\ell ℜ}\left(u_{j}\right)\right)}^{2}+{\left(u_{\alpha }^{uℜ}\left(u_{j}\right)\right)}^{2}+{\left(v_{\alpha }^{\ell ℜ}\left(u_{j}\right)\right)}^{2}+{\left(v_{\alpha }^{uℜ}\left(u_{j}\right)\right)}^{2}+{\left(w_{\alpha }^{\ell ℜ}\left(u_{j}\right)\right)}^{2}+{\left(w_{\alpha }^{uℜ}\left(u_{j}\right)\right)}^{2}\right)}\\ \sqrt{\left({\left(u_{\alpha }^{\ell L}\left(u_{j}\right)\right)}^{2}+{\left(u_{\alpha }^{uL}\left(u_{j}\right)\right)}^{2}+{\left(v_{\alpha }^{\ell L}\left(u_{j}\right)\right)}^{2}+{\left(v_{\alpha }^{uL}\left(u_{j}\right)\right)}^{2}+{\left(w_{\alpha }^{\ell L}\left(u_{j}\right)\right)}^{2}+{\left(w_{\alpha }^{uL}\left(u_{j}\right)\right)}^{2}\right)} \end{array}\right) \end{array}}. \end{array}$$

**Theorem 7 .**

Let *℘*_*ℜ*_, *℘*_*ℒ*_, and *℘*_*Q*_ be three mPIVNSSs. Then, the following properties hold.

1. 0 ≤ *𝒮*_mPIVNSS_^1^(*℘*_*ℜ*_, *℘*_*ℒ*_) ≤ 1
2. *𝒮*_mPIVNSS_^1^(*℘*_*ℜ*_, *℘*_*ℒ*_)=*𝒮*_mPIVNSS_^1^( *℘*_*ℒ*_, *℘*_*ℜ*_)
3. If *℘*_*ℜ*_⊆*℘*_*ℒ*_⊆*℘*_*Q*_, then *𝒮*_mPIVNSS_^1^(*℘*_*ℜ*_, *℘*_*Q*_) ≤ *𝒮*_mPIVNSS_^1^(*℘*_*ℜ*_, *℘*_*ℒ*_) and *𝒮*_mPIVNSS_^1^(*℘*_*ℜ*_, *℘*_*Q*_) ≤ *𝒮*_mPIVNSS_^1^(*℘*_*ℒ*_, *℘*_*Q*_)

**Proof**

By using the above definition, the proof of these properties can be done easily.

**Definition 39 .**

Let *℘*_*R*_1__ and *℘*_*R*_2__ be two mPIVNSSs over the universe of discourse *𝒰*={*u*_1_,  *u*_2_,…,  *u*_*j*_}. Then, the set-theoretic similarity measure between *℘*_*R*_1__ and *℘*_*R*_2__ is defined as

(61) $$\begin{array}{c} S_{\text{mPIVNSS}}^{2}\left({℘}_{R_{1}},{℘}_{R_{2}}\right)=\frac{1}{mn}\overset{n}{\underset{j=1}{\sum }}\overset{m}{\underset{\alpha =1}{\sum }}\frac{\left(\begin{array}{c} \left(u_{\alpha }^{\ell ℜ}\left(u_{j}\right)+u_{\alpha }^{uℜ}\left(u_{j}\right)\right)\left(u_{\alpha }^{\ell L}\left(u_{j}\right)+\;u_{\alpha }^{uL}\left(u_{j}\right)\right)+\left(v_{\alpha }^{\ell ℜ}\left(u_{j}\right)+v_{\alpha }^{uℜ}\left(u_{j}\right)\right)\left(v_{\alpha }^{\ell L}\left(u_{j}\right)+\;v_{\alpha }^{uL}\left(u_{j}\right)\right)+\\ \left(w_{\alpha }^{\ell ℜ}\left(u_{j}\right)+w_{\alpha }^{uℜ}\left(u_{j}\right)\right)\left(w_{\alpha }^{\ell L}\left(u_{j}\right)+\;w_{\alpha }^{uL}\left(u_{j}\right)\right) \end{array}\right)}{\begin{array}{c} \max\left\{\begin{array}{c} \sqrt{\left({\left(u_{\alpha }^{\ell ℜ}\left(u_{j}\right)\right)}^{2}+{\left(u_{\alpha }^{uℜ}\left(u_{j}\right)\right)}^{2}+{\left(v_{\alpha }^{\ell ℜ}\left(u_{j}\right)\right)}^{2}+{\left(v_{\alpha }^{uℜ}\left(u_{j}\right)\right)}^{2}+{\left(w_{\alpha }^{\ell ℜ}\left(u_{j}\right)\right)}^{2}+{\left(w_{\alpha }^{uℜ}\left(u_{j}\right)\right)}^{2}\right)}\\ \sqrt{\left({\left(u_{\alpha }^{\ell L}\left(u_{j}\right)\right)}^{2}+{\left(u_{\alpha }^{uL}\left(u_{j}\right)\right)}^{2}+{\left(v_{\alpha }^{\ell L}\left(u_{j}\right)\right)}^{2}+{\left(v_{\alpha }^{uL}\left(u_{j}\right)\right)}^{2}+{\left(w_{\alpha }^{\ell L}\left(u_{j}\right)\right)}^{2}+{\left(w_{\alpha }^{uL}\left(u_{j}\right)\right)}^{2}\right)} \end{array}\right\} \end{array}}. \end{array}$$

**Theorem 8 .**

Let *℘*_*ℜ*_ and *℘*_*ℒ*_ be two mPIVNSSs over *𝒰*. Then, the following properties hold.

1. 0 0 ≤ *𝒮*_mPIVNSS_^2^(*℘*_*ℜ*_, *℘*_*ℒ*_) ≤ 1
2. *𝒮*_mPIVNSS_^2^(*℘*_*ℜ*_, *℘*_*ℒ*_)=*𝒮*_mPIVNSS_^2^(*℘*_*ℒ*_, *℘*_*ℜ*_)
3. If *℘*_*ℜ*_⊆*℘*_*ℒ*_⊆*℘*_*Q*_, then *𝒮*_mPIVNSS_^2^(*℘*_*ℜ*_, *℘*_*Q*_) ≤ *𝒮*_mPIVNSS_^2^(*℘*_*ℜ*_, *℘*_*ℒ*_) and *𝒮*_mPIVNSS_^2^(*℘*_*ℜ*_, *℘*_*Q*_) ≤ *𝒮*_mPIVNSS_^2^(*℘*_*ℒ*_, *℘*_*Q*_)

**Proof**

By using the above definition, the proof of these properties can be done easily.

## 6. Application of Similarity Measures and Correlation Coefficient of mPIVNSS for Decision Making

In this section, we utilized the developed approaches based on correlation coefficient, mPIVNSWA operator, and similarity measures for decision making.

### 6.1. Numerical Example

A university calls for the appointment of a vacant position of associate professor. For further assessment, four candidates (alternatives) choose after preliminary review such as {*β*^(1)^, *β*^(2)^, *β*^(3)^, *β*^(4)^}. A team of three experts has been hired by the president of the institution {*u*_1_, *u*_2_, *u*_3_} with weights (0.25, 0.30, 0.45)^*T*^ for final scrutiny and provide the selection criteria, as given in Table 1. First of all, the group of experts decides the parameters for the selection of the candidate such as *e*_1_ = experience, *e*_2_ = publications, and *e*_3_ = research quality with weights (0.35, 0.25, 0.40)^*T*^. Each expert gives his preferences for each alternative in the form of mPIVNSNs under the considered parameters given in Tables 2–5. The developed methods to find the best alternative for the position of associate professor are presented in Algorithm 1, Definitions 35 and 36.

### 6.2. Applications of Proposed Approaches

Assume {*β*^(1)^, *β*^(2)^, *β*^(3)^, *β*^(4)^} be a set of alternatives who are shortlisted for interview and *ℰ*={*e*_1_=experience,*e*_2_=publications,*e*_3_=research quality} be a set of parameters for the selection of associate professor. Let *ℜ* and *ℒ*⊆*ℰ*; then, we construct the 3-PIVNSS *℘*_*ℜ*_(*e*) according to the requirement of university management such as follows:

Construct 3-PIVNSS *℘*_*ℒ*_^(*t*)^(*e*) for each alternative according to experts, where *t* = 1, 2, 3, 4.

#### 6.2.1. Solution by Using Algorithm 1

By using Tables 1–5, compute the correlation coefficient between *δ*_mPIVNSS_(*℘*_*ℜ*_(*e*), *℘*_*ℒ*_^(1)^(*e*)), *δ*_mPIVNSS_(*℘*_*ℜ*_(*e*), *℘*_*ℒ*_^(2)^(*e*)), *δ*_mPIVNSS_(*℘*_*ℜ*_(*e*), *℘*_*ℒ*_^(3)^(*e*)), and *δ*_mPIVNSS_(*℘*_*ℜ*_(*e*), *℘*_*ℒ*_^(4)^(*e*)) by using equation (54), such as

(62) $$\begin{array}{c} \delta_{\text{mPIVNSS}}\left({℘}_{ℜ}\left(e\right),{℘}_{L}^{\left(1\right)}\left(e\right)\right)=\left\{\begin{array}{c} \frac{\left(0.3\right)\left(0.2\right)+\left(0.5\right)\left(0.4\right)+\left(0.2\right)\left(0.4\right)+\left(0.4\right)\left(0.5\right)+\left(0.2\right)\left(0.3\right)+\left(0.6\right)\left(0.4\right)}{\sqrt{{\left(0.3\right)}^{2}+{\left(0.5\right)}^{2}+{\left(0.2\right)}^{2}+{\left(0.4\right)}^{2}+{\left(0.2\right)}^{2}+{\left(0.6\right)}^{2}}\;\sqrt{{\left(0.2\right)}^{2}+{\left(0.4\right)}^{2}+{\left(0.4\right)}^{2}+{\left(0.5\right)}^{2}+{\left(0.3\right)}^{2}+{\left(0.4\right)}^{2}}}\\ +\frac{\left(0.2\right)\left(0.6\right)+\left(0.3\right)\left(0.7\right)+\left(0.5\right)\left(0.1\right)+\left(0.7\right)\left(0.2\right)+\left(0.1\right)\left(0.2\right)+\left(0.3\right)\left(0.3\right)}{\sqrt{{\left(0.2\right)}^{2}+{\left(0.3\right)}^{2}+{\left(0.5\right)}^{2}+{\left(0.7\right)}^{2}+{\left(0.1\right)}^{2}+{\left(0.3\right)}^{2}}\sqrt{{\left(0.6\right)}^{2}+{\left(0.7\right)}^{2}+{\left(0.1\right)}^{2}+{\left(0.2\right)}^{2}+{\left(0.2\right)}^{2}+{\left(0.3\right)}^{2}}}\\ +\cdots +\frac{\left(0.2\right)\left(0.2\right)+\left(0.4\right)\left(0.5\right)+\left(0.3\right)\left(0.2\right)+\left(.5\right)\left(.3\right)+\left(.3\right)\left(0.4\right)+\left(0.6\right)\left(0.6\right)}{\sqrt{{\left(0.2\right)}^{2}+{\left(0.4\right)}^{2}+{\left(0.3\right)}^{2}+{\left(0.5\right)}^{2}+{\left(0.3\right)}^{2}+{\left(0.6\right)}^{2}}\sqrt{{\left(0.2\right)}^{2}+{\left(0.5\right)}^{2}+{\left(0.2\right)}^{2}+{\left(0.3\right)}^{2}+{\left(0.4\right)}^{2}+{\left(0.6\right)}^{2}}} \end{array}\right\}=\left(\frac{24.28}{27.7036}\right)=\;0.87642. \end{array}$$

**Figure 3:**
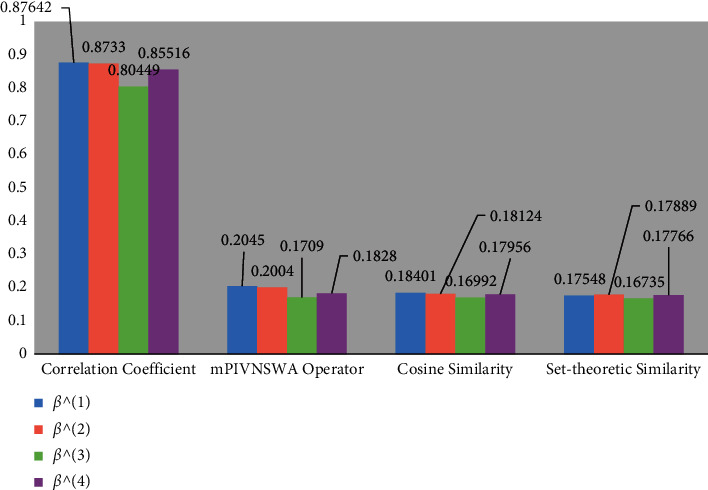
Ranking of alternatives by using proposed techniques.

Similarly, we can find the CC between *δ*_mPIVNSS_(*℘*_*ℜ*_(*e*), *℘*_*ℒ*_^(2)^(*e*)), *δ*_mPIVNSS_(*℘*_*ℜ*_(*e*), *℘*_*ℒ*_^(3)^(*e*)), and *δ*_mPIVNSS_(*℘*_*ℜ*_(*e*), *℘*_*ℒ*_^(4)^(*e*)) given as. *δ*_mPIVNSS_(*℘*_*ℜ*_(*e*), *℘*_*ℒ*_^(2)^(*e*))=(25.04/28.6727)=0.87330, *δ*_mPIVNSS_(*℘*_*ℜ*_(*e*), *℘*_*ℒ*_^(3)^(*e*))=(23.73/29.4968)=0.80449, and *δ*_mPIVNSS_(*℘*_*ℜ*_(*e*), *℘*_*ℒ*_^(4)^(*e*))=(24.58/28.7433)=0.85516. This shows that *δ*_mPIVNSS_(*℘*_*ℜ*_(*e*), *℘*_*ℒ*_^(1)^(*e*)) >  *δ*_mPIVNSS_(*℘*_*ℜ*_(*e*), *℘*_*ℒ*_^(2)^(*e*)) > *δ*_mPIVNSS_(*℘*_*ℜ*_(*e*), *℘*_*ℒ*_^(4)^(*e*))  > *δ*_mPIVNSS_(*℘*_*ℜ*_(*e*), *℘*_*ℒ*_^(3)^(*e*)). The above-obtained ranking shows that *β*^(1)^ is the best alternative. So, the ranking of other alternatives is given as *β*^(1)^ > *β*^(2)^ > *β*^(4)^ > *β*^(3)^. Graphical results are shown in Figure 3.

#### 6.2.2. Solution by Using Algorithm 2

  Step 1: experts evaluate the scores for each alternative in the form of mPIVNSNs given in Tables 2–5.
   Step 2: utilizing equation (59), the opinion of experts for each alternative can be summarized as follows: Δ_1_=[0.3144, 0.5379], [0.1819, 0.3711], [0.2437, 0.3752], Δ_2_=[0.4569, 0.6073], [0.2813, 0.3947], [0.2988, 0.4815], Δ_3_=[0.3303, 0.4884], [0.3018, 0.4429], [0.4296, 0.5670], and Δ_4_=[0.3530, 0.5200], [0.2815, 0.4420], [0.3546, 0.5037].
   Step 3: utilizing equation (60), compute the score values for each alternative. *𝕊*(Δ_1_)=0.2045, *𝕊*(Δ_2_)=0.2004, *𝕊*(Δ_3_)=0.1709, and *𝕊*(Δ_4_)=0.1828.
   Step 4: so, alternatives' ranking is as follows: *𝕊*(Δ_1_) > *𝕊*(Δ_2_) > *𝕊*(Δ_4_) > *𝕊*(Δ_3_). So, *β*^(1)^ > *β*^(2)^  > *β*^(4)^ > *β*^(3)^; hence, the alternative *β*^(1)^ is the most suitable alternative for the position of associate professor. Graphical representation of the obtained results is shown in Figure 3.

#### 6.2.3. Solution by Using Algorithm 3

By using Tables 1–5, compute the cosine similarity measure between *δ*_mPIVNSS_^1^(*℘*_*ℜ*_(*e*), *℘*_*ℒ*_^(1)^(*e*)), *δ*_mPIVNSS_^1^(*℘*_*ℜ*_(*e*), *℘*_*ℒ*_^(2)^(*e*)), *δ*_mPIVNSS_^1^(*℘*_*ℜ*_(*e*), *℘*_*ℒ*_^(3)^(*e*)), and *δ*_mPIVNSS_^1^(*℘*_*ℜ*_(*e*), *℘*_*ℒ*_^(4)^(*e*)) by using equation (60), such as

(63) $$\begin{array}{c} \delta_{\text{mPIVNSS}}^{1}\left({℘}_{ℜ}\left(e\right),{℘}_{L}^{\left(1\right)}\left(e\right)\right)=\frac{1}{3\times 3}\left\{\begin{array}{c} \frac{\left(0.8\right)\left(0.6\right)+\left(0.6\right)\left(0.9\right)+\left(0.8\right)\left(0.7\right)}{\sqrt{{\left(0.3\right)}^{2}+{\left(0.5\right)}^{2}+{\left(0.2\right)}^{2}+{\left(0.4\right)}^{2}+{\left(0.2\right)}^{2}+{\left(0.6\right)}^{2}}\;\sqrt{{\left(0.2\right)}^{2}+{\left(0.4\right)}^{2}+{\left(0.4\right)}^{2}+{\left(0.5\right)}^{2}+{\left(0.3\right)}^{2}+{\left(0.4\right)}^{2}}}\\ +\frac{\left(0.5\right)\left(1.3\right)+\left(1.2\right)\left(0.3\right)+\left(0.4\right)\left(0.5\right)}{\sqrt{{\left(0.2\right)}^{2}+{\left(0.3\right)}^{2}+{\left(0.5\right)}^{2}+{\left(0.7\right)}^{2}+{\left(0.1\right)}^{2}+{\left(0.3\right)}^{2}}\sqrt{{\left(0.6\right)}^{2}+{\left(0.7\right)}^{2}+{\left(0.1\right)}^{2}+{\left(0.2\right)}^{2}+{\left(0.2\right)}^{2}+{\left(0.3\right)}^{2}}}+\cdots \\ +\frac{\left(0.6\right)\left(0.6\right)+\left(0.8\right)\left(0.8\right)+\left(0.9\right)\left(9\right)}{\sqrt{{\left(0.2\right)}^{2}+{\left(0.4\right)}^{2}+{\left(0.3\right)}^{2}+{\left(0.5\right)}^{2}+{\left(0.3\right)}^{2}+{\left(0.6\right)}^{2}}\sqrt{{\left(0.2\right)}^{2}+{\left(0.5\right)}^{2}+{\left(0.2\right)}^{2}+{\left(0.3\right)}^{2}+{\left(0.4\right)}^{2}+{\left(0.6\right)}^{2}}} \end{array}\right\}=\frac{1}{9}\left(\frac{45.88}{27.7036}\right)=0.18401. \end{array}$$

Similarly, we can find the cosine similarity measure between *δ*_mPIVNSS_^1^(*℘*_*ℜ*_(*e*), *℘*_*ℒ*_^(2)^(*e*)), *δ*_mPIVNSS_^1^(*℘*_*ℜ*_(*e*), *℘*_*ℒ*_^(3)^(*e*)), and *δ*_mPIVNSS_^1^(*℘*_*ℜ*_(*e*), *℘*_*ℒ*_^(4)^(*e*)) given as *δ*_mPIVNSS_^1^(*℘*_*ℜ*_(*e*), *℘*_*ℒ*_^(2)^(*e*))=(1/9)(46.77/28.6727)=0.18124, *δ*_mPIVNSS_^1^(*℘*_*ℜ*_(*e*), *℘*_*ℒ*_^(3)^(*e*))=(1/9)(45.11/29.4968)= 0.16992, and *δ*_mPIVNSS_^1^(*℘*_*ℜ*_(*e*), *℘*_*ℒ*_^(4)^(*e*))=(1/9)(46.45/28.7433)=0.17956. This shows that *δ*_mPIVNSS_^1^(*℘*_*ℜ*_(*e*), *℘*_*ℒ*_^(1)^(*e*)) >  *δ*_mPIVNSS_^1^(*℘*_*ℜ*_(*e*), *℘*_*ℒ*_^(2)^(*e*)) > *δ*_mPIVNSS_^1^(*℘*_*ℜ*_(*e*), *℘*_*ℒ*_^(4)^(*e*)) > *δ*_mPIVNSS_^1^(*℘*_*ℜ*_(*e*), *℘*_*ℒ*_^(3)^(*e*)), which shows that alternative *β*^(1)^ is the most appropriate and similar to *℘*_*ℜ*_(*e*). So, alternatives ranking is given as *β*^(1)^ > *β*^(2)^  > *β*^(4)^ > *β*^(3)^.

Now, we compute the set-theoretic similarity measure by using Definition 37 between *δ*_mPIVNSS_^2^(*℘*_*ℜ*_(*e*), *℘*_*ℒ*_^(1)^(*e*)), *δ*_mPIVNSS_^2^(*℘*_*ℜ*_(*e*), *℘*_*ℒ*_^(2)^(*e*)), *δ*_mPIVNSS_^2^(*℘*_*ℜ*_(*e*), *℘*_*ℒ*_^(3)^(*e*)), and *δ*_mPIVNSS_^2^(*℘*_*ℜ*_(*e*), *℘*_*ℒ*_^(4)^(*e*)). From Tables 1–5, we can find the set-theoretic similarity measure for each alternative by using equation (61) given as *δ*_mPIVNSS_^2^(*℘*_*ℜ*_(*e*), *℘*_*ℒ*_^(1)^(*e*))=0.17889, *δ*_mPIVNSS_^2^(*℘*_*ℜ*_(*e*), *℘*_*ℒ*_^(2)^(*e*))=0.17548, *δ*_mPIVNSS_^2^(*℘*_*ℜ*_(*e*), *℘*_*ℒ*_^(3)^(*e*))=0.16735, and *δ*_mPIVNSS_^2^(*℘*_*ℜ*_(*e*), *℘*_*ℒ*_^(4)^(*e*))=0.17766. This shows that *δ*_mPIVNSS_^2^(*℘*_*ℜ*_(*e*), *℘*_*ℒ*_^(1)^(*e*))  > *δ*_mPIVNSS_^2^(*℘*_*ℜ*_(*e*), *℘*_*ℒ*_^(4)^(*e*)) > *δ*_mPIVNSS_^2^(*℘*_*ℜ*_(*e*), *℘*_*ℒ*_^(2)^(*e*)) > *δ*_mPIVNSS_^2^(*℘*_*ℜ*_(*e*), *℘*_*ℒ*_^(3)^(*e*)). So, *β*^(1)^ is the best alternative using the set-theoretic similarity measure, and the ranking of other alternatives is given as *β*^(1)^ > *β*^(4)^  > *β*^(2)^ > *β*^(3)^. Graphical representation of results is shown in Figure 3.

## 7. Discussion and Comparative Analysis

In the next section, we are going to talk about utility, ease, and management with the help of a planned method. We also made a tentative assessment of the following with planned techniques and some existing methods.

### 7.1. Superiority and Advantage of the Proposed Method

Through this study and comparison, it can be determined that the results obtained from the proposed approach are either more general than the methods available. Although, on the whole, the DM method associated with the usual DM methods adjusts the additional information to overcome the hesitation. Also, the various hybrid structures of FS are becoming a special feature of mPIVNS, with some suitable conditions being added. General information related to the object can be described accurately and analytically, as given in Table 6. Therefore, the proposed approach precedes the specific hybrid structure of the practical, modest, and fuzzy set.

It turns out to be a contemporary problem. Why do we have to particularize novel algorithms according to the present novel structure? There are several indications that the recommended methodology can be exceptional compared to other existing methods. We remember the fact that IFS, picture fuzzy set, FS, hesitant fuzzy set, NS, and other fuzzy sets have been restricted by the mixed structure and cannot provide complete information regarding the situation. But, the proposed model in this study be the utmost appropriate for MCDM because it can handle three types of information such as truth, falsity, and indeterminacy. Comparative analysis with some common methods is given in Table 6. So, the established model is multipurpose and can simply resolve problems comparative to intuitionistic, neutrosophy, hesitation, image, and ambiguity substitution. Hence, we claim that the presented similarity measures and other developed measures for mPIVNSS deliver the most appropriate information.

### 7.2. Discussion

Chen et al.' [44] multipolar information of fuzzy sets deal with the membership value of the objects, and a multipolar fuzzy set is unable to handle the circumstances when the objects have indeterminacy and falsity information. Xu et al. [52] and Zhang et al.' [53] IFS only deal with the membership and nonmembership values of the alternatives, and these techniques are unable to deal with the multipolar information and indeterminacy of the alternative. Yager [55, 56] and Naeem et al.' [57] PFS and mPyFS cannot handle the indeterminacy of the alternatives. Comparative to the abovementioned theories, our established technique delivers more efficient outcomes for the MCDM problem. The method of Zhang et al. [54] and Ali et al. [46] dealt with the truthiness, indeterminacy, and falsity grades for alternatives, but these techniques cannot manage multiple data. Instead, our established approach is an innovative method that can cope with a wide variety of information alternatives. A comparison is given in Table 6. Meanwhile, the developed method handles truth, indeterminacy, and falsity of alternatives. Thus, our developed method is extracompetent and delivers well outcomes for decision-makers over extra data.

### 7.3. Comparative Analysis

We recommend some novel algorithms under mPIVNSS by utilizing the developed mPIVNSS such as the mPIVNSWA operator, correlation coefficient, and similarity measures in the following section. Subsequently, we utilize the suggested algorithms to a realistic problem, namely, for the selection of an appropriate associate professor. It can be observed that *β*^(1)^ is the finest alternative for the position of associate professor. The proposed approach can be compared to other available methods and observed that our proposed methodologies deliver the most reliable results comparative to available techniques. We observe one most interesting fact in our obtained results that our proposed methodologies deliver the same optimal and worst choices. The comparison of our proposed methodologies with some existing approaches is given in Table 7.

The research concludes that the results obtained from the planned point of view exceed the results of the prevailing theories. Therefore, compared to existing techniques, established AOs, similarity measures, and CC handled uncertain and confusing information efficiently. However, under the current DM strategy, the main advantage of the planned method is that it can accommodate additional information in the data compared to existing techniques. This is a useful tool for resolving misinformation and vagueness in the DM method. The advantage of a planned approach with measures related to the current approach is avoiding the consequences based on negative reasons.

## 8. Conclusion

In this study, a novel hybrid structure has been established by merging two independent structures m-polar fuzzy set and interval-valued neutrosophic soft set which is known as mPIVNSS. Some fundamental operations with their properties have been introduced for mPIVNSS. We have developed the CC and WCC with their properties in the content of mPIVNSS and also defined some operational laws for mPIVNSS and established a novel operator such as m-polar interval-valued neutrosophic weighted aggregation operator based on developed operational laws. To compute the similarity measure between two mPIVNSS, the idea of cosine and set-theoretic similarity measures have been established. Three novel algorithms based on mPIVNSS have been constructed to solve MCDM problems, correlation coefficient, mPIVNSWA operator, and similarity measures. A comparative analysis was also performed to demonstrate the proposed method. Finally, the projected ideas presented high constancy and functionality for decision-makers in the decision-making process. Based on the results acquired, it has been terminated and the above approach is extremely appropriate for finding the problem of MCDM in today's life. In the future, anyone can be introduced to the multipolar interval-valued neutrosophic weighted geometric operator with its decision-making approach. Furthermore, the concept of mPIVNSS will be extended to a multipolar interval-valued neutrosophic hypersoft set with their basic operators. The proposed impression can be functional to moderately a lot of problems in real life, including the therapeutic career, computing, artificial intelligence, pattern recognition, and finances.

## Figures and Tables

**Algorithm 1:**
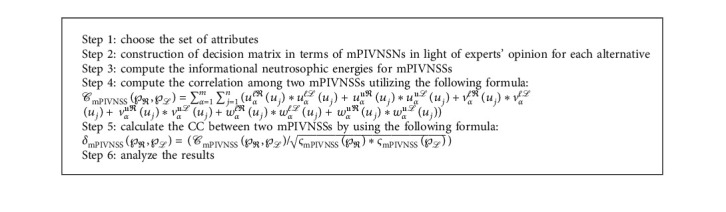
For the correlation coefficient of mPIVNSS.

**Algorithm 2:**
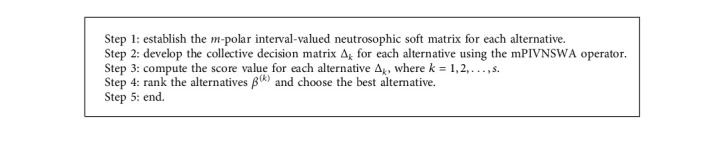
For mPIVNSWA operator.

**Algorithm 3:**
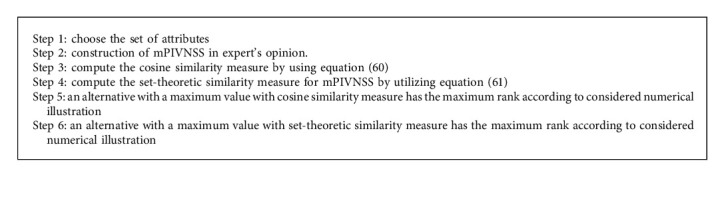
For similarity measure of mPIVNSS.

**Table 1:** Construction of 3-PIVNSS of alternatives according to management requirement.

*℘*_*R*_(**e**)	**e**_1_	**e**_2_	**e**_3_
**u**_1_	([0.3, 0.5], [0.2, 0 .4], [0.2, 0.6]), ([0.2, 0.3], [0.5, 0.7], [0.1, 0.3]), ([0.5, .6], [0.1, 0.3], [0.4, 0.6])	([0.2, 0.4], [0.3, 0.5], [0.3, 0.6]), ([0.2, 0.3], [0.2, 0.4], [0.4, 0.5]), ([0.4, 0.6], [0.1, 0.3], [0.2, 0.4])	([0.6, 0.7], [0.2, 0.3], [0.3, 0.4]), ([0.4, 0.5], [0.5, 0.8], [0.1, 0.2]), ([0.1, 0.2], [0.5, 0.8], [0.2, 0.4])
**u**_2_	([0.5, 0.7], [0.1, 0.2], [0.4, 0.6]), ([0.2, 0.4], [0.3, 0.4], [0.2, 0.5]), ([0.6, 0.8], [0.1, 0.2], [0.3, 0.5])	([0.5, 0 .6], [0.2, 0.3], [0.3, 0.4]), ([0.3, 0.5], [0.4, 0.5], [0.1, 0.3]), ([0.4, 0.6], [0.4, 0.5], [0.3, 0.5])	([0.5, 0.7], [0.1, 0.2], [0.5, 0.6]), ([0.2, 0.4], [0.5, 0.6], [0.4, 0.6]), ([0.2, 0.4], [0.3, 0.4], [0.2, 0.5])
**u**_3_	([0.4, 0.6], [0.2, 0 .3], [0.1, 0.4]), ([0.2, 0 .5], [0.2, 0.3], [0.1, 0.6]), ([0.3, 0.4], [0.2, 0.5], [0.5, 0.7])	([0.3, 0.5], [0.4, 0.5], [0.1, 0.3]), ([0.2, 0.4], [0.7, 0.8], [0.1, 0.2]), ([0.1, 0.2], [0.7, .8], [0.2, 0.3]])	([0.2, 0.3], [0.5, 0.7], [0.1, 0.3]), ([0.3, 0.4], [0.2, 0.5], [0.5, 0.7]), ([0.2, 0.4], [0.3, 0.5], [0.3, 0.6])

**Table 2:** Evaluation report for alternative *β*^(1)^.

*℘*_*ℒ*_^(1)^(**e**)	**e**_1_	**e**_2_	**e**_3_
**u**_1_	([0.2, 0.4], [0.4, 0.5], [0.3, 0.4]), ([0.6, 0.7], [0.1, 0.2], [0.2, 0.3]), ([0.3, 0.4], [0.4, 0.5], [0.2, 0.4])	([0.3, 0.4], [0.4, 0.5], [0.2, 0.5]), ([0.3, 0.6], [0.2, 0.3], [0.1, 0.2]), ([0.4, 0.6], [0.2, 0.3], [0.4, 0.5])	([0.2, 0.4], [0.4, 0.6], [0.1, 0.2]), ([0.1, 0.3], [0.6, 0.7], [0.2, 0.3]), ([0.4, 0.5], [0.2, 0.6], [0.2, 0.3])
**u**_2_	([0.5, 0.7], [0.1, 0.2], [0.2, 0.4]), ([0.7, 0.8], [0.1, 0.2], [0.2, 0.4]), ([0.1, 0.3], [0.1, 0.5], [0.2, 0.5])	([0.1, 0.4], [0.2, 0.4], [0.1, 0.2]), ([0.2, 0.5], [0.2, 0.4], [0.3, 0.5]), ([0.3, 0.5], [0.2, 0.4], [0.4, 0.6])	([0.5, 0.7], [0.1, 0.2], [0.5, 0.6]), ([0.3, 0.5], [0.3, 0.4], [0.6, 0.7]), ([0.2, 0.4], [0.3, 0.4], [0.2, 0.5])
**u**_3_	([0.4, 0.5], [0.2, 0.5], [0.1, 0.2]), ([0.4, 0.7], [0.1, 0.2], [0.1, 0.2]), ([0.3, 0.4], [0.2, 0.5], [0.5, 0.7])	([0.6, 0.8], [0.1, 0.2], [0.1, 0.5]), ([0.2, 0.4], [0.7, 0.8], [0.1, 0.2]), ([0.5, 0.7], [0.1, 0.2], [0.2, 0.4])	([0.5, 0.6], [0.2, 0.3], [0.4, 0.5]), ([0.3, 0.4], [0.4, 0.5], [0.2, 0.4]), ([0.2, 0.4], [0.3, 0.5], [0.3, 0.6])

**Table 3:** Evaluation report for alternative *β*^(2)^.

*℘*_*ℒ*_^(2)^(**e**)	**e**_1_	**e**_2_	**e**_3_
**u**_1_	([0.2, 0.4], [0.4, 0.6], [0.4, 0.5]), ([0.2, 0.3], [0.4, 0.6], [0.3, 0.5]), ([0.1, 0.2], [0.6, 0.8], [0.2, 0.5])	([0.4, 0.5], [0.2, 0.5], [0.1, 0.2]), ([0.2, 0.3], [0.4, 0.6], [0.3, 0.5]), ([0.1, 0.2], [0.6, 0.8], [0.2, 0.5])	([0.7, 0.8], [0.1, 0.2], [0.2, 0.3]), ([0.1, 0.3], [0.6, 0.7], [0.2, 0.5]), ([0.4, 0.5], [0.2, 0.5], [0.1, 0.2])
**u**_2_	([0.5, 0.7], [0.1, 0.2], [0.2, 0.4]), ([0.1, 0.3], [0.5, 0.7], [0.2, 0.6]), ([0.1, 0.4], [0.2, 0.5], [0.4, 0.6])	([0.1, 0.4], [0.2, 0.4], [0.1, 0.2]), ([0.1, 0.2], [0.2, 0.5], [0.4, 0.6]), ([0.1, 0.4], [0.2, 0.5], [0.4, 0.6])	([0.1, 0.4], [0.2, 0.5], [0.4, 0.6]), ([0.3, 0.4], [0.2, 0.6], [0.4, 0.6]), ([0.2, 0.4], [0.3, 0.4], [0.2, 0.5])
**u**_3_	([0.4, 0.5], [0.2, 0.5], [0.1, 0.2]), ([0.1, 0.2], [0.2, 0.5], [0.4, 0.6]), ([0.3, 0.5], [0.3, 0.5], [0.6, 0.7])	([0.3, 0.5], [0.3, 0.5], [0.6, 0.7]), ([0.1, 0.2], [0.2, 0.5], [0.4, 0.6]), ([0.5, 0.7], [0.1, 0.2], [0.2, 0.4])	([0.2, 0.4], [0.4, 0.5], [0.6, 0.8]), ([0.3, 0.5], [0.3, 0.5], [0.6, 0.7]), ([0.1, 0.2], [0.2, 0.5], [0.4, 0.6])

**Table 4:** Evaluation report for alternative *β*^(3)^.

*℘*_*ℒ*_^(3)^(**e**)	**e**_1_	**e**_2_	**e**_3_
**u**_1_	([0.6, 0.7], [0.1, 0.2], [0.3, 0.5]), ([0.6, 0.8], [0.1, 0.2], [0.2, 0.3]), ([0.6, 0.7], [0.3, 0.5], [0.1, 0.2])	([0.7, 0.8], [0.1, 0.2], [0.2, 0.5]), ([0.6, 0.7], [0.1, 0.2], [0.1, 0.2]), ([0.5, 0.8], [0.1, 0.2], [0.2, 0.4])	([0.1, 0.3], [0.6, 0.7], [0.2, 0.5]), ([0.7, 0.8], [0.1, 0.2], [0.2, 0.3]), ([0.5, 0.7], [0.3, 0.4], [0.2, 0.3])
**u**_2_	([0.5, 0.7], [0.2, 0.5], [0.2, 0.3]), ([0.7, 0.8], [0.3, 0.5], [0.1, 0.3]), ([0.4, 0.7], [0.2, 0.3], [0.3, 0.7])	([0.5, 0.6], [0.3, 0.4], [0.1, 0.2]), ([0.1, 0.2], [0.2, 0.5], [0.4, 0.6]), ([0.4, 0.6], [0.2, 0.3], [0.1, 0.2])	([0.1, 0.4], [0.2, 0.5], [0.4, 0.6]), ([0.4, 0.6], [0.2, 0.3], [0.1, 0.2]), ([0.2, 0.4], [0.3, 0.4], [0.2, 0.5])
**u**_3_	([0.4, 0.6], [0.2, 0.3], [0.1, 0.2]), ([0.1, 0.2], [0.2, 0.5], [0.4, 0.6]), ([0.6, 0.8], [0.1, 0.2], [0.1, 0.3])	([0.3, 0.5], [0.3, 0.5], [0.6, 0.7]), ([0.6, 0.8], [0.1, 0.2], [0.1, 0.2]), ([0.7, 0.8], [0.1, 0.2], [0.2, 0.4])	([0.6, 0.8], [0.3, 0.4], [0.1, 0.2]), ([0.5, 0.7], [0.1, 0.2], [0.4, 0.5]), ([0.1, 0.2], [0.2, 0.5], [0.4, 0.6])

**Table 5:** Evaluation report for alternative *β*^(4)^.

*℘*_*ℒ*_^(4)^(**e**)	**e**_1_	**e**_2_	**e**_3_
**u**_1_	([0.3, 0.5], [0.2, 0.4], [0.1, 0.2]), ([0.3, 0.6], [0.1, 0.2], [0.4, 0.7]), ([0.4, 0.7], [0.3, 0.4], [0.2, 0.3])	([0.7, 0.8], [0.2, 0.4], [0.3, 0.5]), ([0.5, 0.7], [0.3, 0.4], [0.2, 0.4]), ([0.4, 0.6], [0.2, 0.5], [0.3, 0.4])	([0.2, 0.3], [0.5, 0.7], [0.2, 0.4]), ([0.5, 0.7], [0.2, 0.4], [0.3, 0.5]), ([0.4, 0.5], [0.5, 0.7], [0.2, 0.4])
**u**_2_	([0.4, 0.7], [0.3, 0.5], [0.2, 0.4]), ([0.5, 0.8], [0.3, 0.6], [0.2, 0.3]), ([0.4, 0.6], [0.2, 0.3], [0.3, 0.5])	([0.5, 0.8], [0.3, 0.4], [0.2, 0.3]), ([0.2, 0.4], [0.2, 0.3], [0.4, 0.5]), ([0.3, 0.5], [0.2, 0.3], [0.3, 0.5])	([0.2, 0.4], [0.2, 0.3], [0.3, 0.6]), ([0.4, 0.6], [0.2, 0.3], [0.1, 0.2]), ([0.2, 0.4], [0.3, 0.4], [0.2, 0.5])
**u**_3_	([0.3, 0.5], [0.3, 0.5], [0.1, 0.2]), ([0.1, 0.2], [0.2, 0.5], [0.4, 0.6]), ([0.5, 0.7], [0.2, 0.4], [0.1, 0.3])	([0.3, 0.5], [0.4, 0.6], [0.6, 0.7]), ([0.5, 0.7], [0.1, 0.2], [0.4, 0.5]), ([0.3, 0.5], [0.2, 0.5], [0.1, 0.3])	([0.4, 0.6], [0.3, 0.5], [0.1, 0.2]), ([0.6, 0.7], [0.1, 0.2], [0.3, 0.5]), ([0.2, 0.5], [0.2, 0.3], [0.4, 0.6])

**Table 6:** Sensitivity analysis of the proposed approach with existing techniques.

	Set	Truthiness	Indeterminacy	Falsity	Multipolarity	Loss of information
Chen et al. [44]	mPFS	✓	×	×	✓	×
Xu et al. [52]	IFS	✓	×	✓	×	×
Zhang et al. [53]	IFS	✓	×	✓	×	✓
Talebi et al. [54]	mPIVIFS	✓	×	✓	✓	✓
Yager [55, 56]	PFS	✓	×	✓	×	×
Naeem et al. [57]	mPyFS	✓	×	✓	✓	×
Zhang et al. [54]	INSs	✓	✓	✓	×	×
Ali et al. [46]	BPNSS	✓	✓	✓	×	×
Proposed approach	mPIVNSS	✓	✓	✓	✓	×

**Table 7:** Comparative analysis between the proposed model and existing techniques.

Method	Alternative final ranking	Optimal choice
Saeed et al. [36]	*β* ^(1)^ > *β*^(2)^ > *β*^(4)^ > *β*^(3)^	*β* ^(1)^
Hashmi et al. [19]	*β* ^(1)^ > *β*^(4)^ > *β*^(3)^ > *β*^(2)^	*β* ^(1)^
Zulqarnain et al. [16]	*β* ^(1)^ > *β*^(2)^ > *β*^(4)^ > *β*^(3)^	*β* ^(1)^
Naeem et al. [57]	*β* ^(1)^ > *β*^(3)^ > *β*^(4)^ > *β*^(2)^	*β* ^(1)^
Correlation coefficient	*β* ^(1)^ > *β*^(2)^ > *β*^(4)^ > *β*^(3)^	*β* ^(1)^
mPIVNSWA	*β* ^(1)^ > *β*^(2)^ > *β*^(4)^ > *β*^(3)^	*β* ^(1)^
Cosine similarity measure	*β* ^(1)^ > *β*^(2)^ > *β*^(4)^ > *β*^(3)^	*β* ^(1)^
Set-theoretic similarity measure	*β* ^(1)^ > *β*^(4)^ > *β*^(2)^ > *β*^(3)^	*β* ^(1)^

## Data Availability

No data are used to support this research.
